# Trends in Exposure to Secondhand Smoke Among Adolescents in China From 2013-2014 to 2019: Two Repeated National Cross-sectional Surveys

**DOI:** 10.2196/40782

**Published:** 2023-03-24

**Authors:** Chuanwei Ma, Yayang Huang, Sixuan Li, Min Zhao, Xinying Zeng, Xinbo Di, Costan G Magnussen, Bo Xi, Shiwei Liu

**Affiliations:** 1 Department of Epidemiology, School of Public Health Qilu Hospital, Cheeloo College of Medicine Shandong University Jinan China; 2 Health Education Institute Beijing Center for Disease Prevention and Control Beijing China; 3 Institute of Child and Adolescent Health School of Public Health Peking University Beijing China; 4 Department of Chronic and Noncommunicable Disease Control and Prevention Ningbo Municipal Center for Disease Control and Prevention Ningbo China; 5 Tobacco Control Office Chinese Center for Disease Control and Prevention Beijing China; 6 Department of Nutrition and Food Hygiene School of Public Health, Cheeloo College of Medicine Shandong University Jinan China; 7 Baker Heart and Diabetes Institute Victoria Australia; 8 Research Centre of Applied and Preventive Cardiovascular Medicine University of Turku Turku Finland; 9 Centre for Population Health Research University of Turku and Turku University Hospital Turku Finland

**Keywords:** secondhand smoke exposure, trends, adolescents, China, secondhand smoke, youth

## Abstract

**Background:**

It is well-known that secondhand smoke exposure in childhood or adolescence is positively associated with morbidity and mortality. However, less is known about the current status of and most recent trends in secondhand smoke exposure among adolescents in China.

**Objective:**

We aimed to assess recent changes in the prevalence of secondhand smoke exposure among adolescents in China using nationally representative data.

**Methods:**

We used data from 2 repeated national cross-sectional surveys conducted in 2013-2014 and 2019. A total of 155,117 students (median age 13.5 years) in 2013-2014 and 147,270 students (median age 13.1 years) in 2019 were included in this study. Sociodemographic factors and secondhand smoke exposure information were collected via a standardized questionnaire. Exposure was defined as secondhand smoke exposure ≥1 day during the past 7 days at home or in public places. Other frequencies of secondhand smoke exposure (ie, ≥3 days, ≥5 days, and every day) during the past 7 days were also assessed. The weighted prevalence of secondhand smoke exposure was calculated according to the complex sample design for surveys.

**Results:**

The prevalence of secondhand smoke exposure in any place (home or public places ≥1 day during the past 7 days) decreased from 2013-2014 (72.9%, 95% CI 71.5%-74.3%) to 2019 (63.2%, 95% CI 62%-64.5%), as did exposure at home (2013-2014: 44.4%, 95% CI 43.1%-45.7%; 2019: 34.1%, 95% CI 33.1%-35.2%) and in public places (2013-2014: 68.3%, 95% CI 66.9%-69.6%; 2019: 57.3%, 95% CI 56%-58.6%). The prevalence of secondhand smoke exposure decreased with increased gross domestic product per capita in each of the 2 survey years irrespective of exposure frequency or location. The prevalence of exposure at other frequencies (ie, ≥3 days, ≥5 days, or every day during the past 7 days) also decreased in any place, at home, and in public places. Secondhand smoke exposure was associated with higher school grade level (ninth vs seventh grade: odds ratio [OR] 1.76, 95% CI 1.68-1.84), gender (boys vs girls: OR 1.18, 95% CI 1.15-1.22), urban status (urban vs rural: OR 1.10, 95% CI 1.01-1.19), and cigarette smoking (yes vs no: OR 6.67, 95% CI 5.83-7.62).

**Conclusions:**

Although the prevalence of secondhand smoke exposure among Chinese adolescents declined from 2013-2014 to 2019, it remains unacceptably high. More effective strategies and stronger action are needed in China to further, and dramatically, curb secondhand smoke exposure among adolescents.

## Introduction

### Background

Tobacco products, especially combustible ones, are harmful not only to users’ health but also the health of nonsmokers who are exposed to secondhand smoke [1]. The World Health Organization (WHO) reports that exposure to secondhand smoke leads to 1.2 million deaths annually [2]. There is no safe or risk-free level of secondhand smoke exposure for children and adolescents [3], and more than 65,000 children die each year from diseases related to secondhand smoke exposure [2]. Secondhand smoke exposure occurs in various environments (at home or in any public place) and causes short-term damage in childhood (eg, slowed lung growth, worsened asthma, respiratory infections, and sudden infant death syndrome) [1], a long-term burden of cardiovascular disease [4], and mortality in adulthood [5].

China has the largest number of tobacco consumers in the world. Although the prevalence of current tobacco smoking decreased from 1990 to 2019 (males: –18.2%; females: –20.9%) among individuals aged 15 years or older in China, the prevalence of current tobacco smoking remained high in 2019 (males: 49.7%; females: 3.54%) [6]. Secondhand smoke exposure remains a serious public health concern in China despite evidence that has suggested the prevalence of secondhand smoke exposure in the workplace declined from 55.2% in 2010 to 45.3% in 2015 among Chinese nonsmokers aged 16 to 60 years [7]. In addition, national data from 2013 to 2016 showed that 41.7% (95% CI 40.9%-42.3%) of Chinese youth aged 6 to 17 years were exposed to secondhand smoke at home ≥1 day during the past 7 days [8]. This prevalence of secondhand smoke exposure among Chinese adolescents was higher than the global average level (72.9% vs 62.9%, respectively) [9].

Although considerable work on tobacco control has been undertaken in China in recent years, including the protection of teenagers from secondhand smoke exposure [10,11], there are no contemporary data on the prevalence of secondhand smoke exposure among adolescents in this country. Moreover, most previous studies only reported the prevalence of secondhand smoke exposure at home and public places in general [9,12,13]; no studies were able to precisely categorize the types of public places where exposure occurs. Such data are needed to monitor and guide effective interventions to protect adolescents from secondhand smoke exposure.

### Objectives

Using repeated nationally representative data, we aimed to assess trends in the prevalence of secondhand smoke exposure between 2013-2014 and 2019 according to different locations (at home, in closed public places, in open public places, and on public transportation).

## Methods

### Study Participants

We used repeated cross-sectional data on secondhand smoke exposure collected from the Global Youth Tobacco Survey (GYTS)-China, which was a part of the GYTS conducted in China from October 1, 2013, to April 1, 2014, and the China National Youth Tobacco Survey (NYTS), conducted from May 1, 2019, to December 31, 2019. Each of these 2 national surveys was a national, representative, school-based, cross-sectional survey administered to middle school students. Both surveys used similar sampling, questionnaires, and survey methodologies and covered 31 provinces, autonomous regions, and municipalities (hereafter referred to as “provinces”) in China. Both surveys used the same 3-stage stratified cluster random sampling strategy. In the first stage, surveillance points were randomly selected from each province, stratified by urban-rural area by probability proportional to size sampling proportional to population size, to be nationally representative, with a total of 336 surveillance points in 2013-2014 and 347 surveillance points in 2019. In the second stage, 3 middle schools were randomly selected at each surveillance point. In the third stage, 1 class was randomly selected from each grade of the selected school. All students in the selected class were eligible to participate. The median age of the study population was 13.5 years in 2013-2014 and 13.1 years in 2019.

### Definition of Secondhand Smoke Exposure

Exposure to secondhand smoke at home was assessed with the question “During the past 7 days, on how many days have people smoked in your presence, in your home?”; exposure in closed public places was assessed with the question “During the past 7 days, on how many days have people smoked in your presence in closed public places other than in your home (such as school buildings, conference halls, gymnasiums, bars, shops, restaurants, shopping malls, movie theaters, etc.)?”; exposure in open public places was assessed with the question “During the past 7 days, on how many days have people smoked in your presence in open public places (such as playgrounds, sidewalks, entrances to buildings, parks, bus stops, parks, etc.)?”; exposure on public transportation was assessed with the question “During the past 7 days, on how many days have people smoked in your presence in any public transportation vehicles (such as trains, buses, or taxicabs, etc.)?” In addition to reporting on secondhand smoke exposure in the individual public places mentioned above, we also unified heterogeneous information by combining exposure that was reported in closed public places, in open public places, and on public transportation together as one variable: “public places.” Moreover, we also combined exposure at home and public places as “any place.” Exposure to secondhand smoke at any location (ie, at home, in closed public places, in open public places, on public transportation, in any public place, or in any place) was defined as exposure to secondhand smoke on ≥1 day during the past 7 days. We also assessed different frequencies (≥3 days, ≥5 days, and every day during the past 7 days) of secondhand smoke exposure based on the students’ responses. To ensure each participant understood that the question was asked about secondhand smoke from others, rather than from themselves, each question was explained by the trained investigators beforehand.

Cigarette smoking was defined as smoking on ≥1 day during the past 30 days with the question “During the past 30 days, on how many days did you smoke cigarettes?” [14]. Exposure to secondhand smoke in schools was assessed with the question “During the past 30 days, did you see anyone smoke inside the school building or outside on school property?” Attitude on the hazards of secondhand smoke exposure was assessed with the question “Do you think the smoke from other people’s cigarettes smoking is harmful to you?” with the corresponding answers “Definitely not, probably not, probably yes, and definitely yes.” Participants were classified into groups based on whether the gross domestic product (GDP) per capita of their province was low, middle, or high. For 2013-2014 (when the exchange rate was US $1=RMB 6.08), the GDP categories were RMB <35,000, RMB 35,000 to 50,000, and RMB >50,000, respectively. For 2019 (when the exchange rate was US $1=RMB 6.98), the categories were RMB <50,000, RMB 50,000 to 70,000, and RMB >70,000, respectively. Further details of the GDP per capita in 2013 and 2019 in China are available online [15,16].

### Ethical Considerations

Both surveys (2013-2014 and 2019) were approved by the National Health Commission, and the proposal and protocols were reviewed by the Institutional Review Board of the Chinese Center for Disease Control and Prevention (202008). All participating students gave oral informed consent. Our study did not contain any identifiable information from individual participants.

### Statistical Analysis

The national, regional, and subgroup (by sex, residence, GDP per capita category, and cigarette smoking status) prevalence with the 95% CI of secondhand smoke exposure in 2013-2014 and 2019 among adolescents was calculated with consideration of sampling weights, strata, and primary sampling units from each survey. The absolute change rate with 95% CI in the prevalence of secondhand smoke exposure was calculated as the prevalence in 2013-2014 subtracted from the prevalence in 2019. The chi-square trend test was used to compare differences in prevalence by school grade, sex, residence, region, GDP per capita category, and cigarette smoking status between 2013-2014 and 2019. Multivariable logistic regression models were used to assess the association between secondhand smoke exposure and its potential influencing factors (school grade, sex, residence, region, GDP per capita category, and cigarette smoking status). All analyses were conducted using SAS (SAS Institute). Two-sided *P* values <.05 were considered statistically significant.

## Results

### Participant Characteristics

Table 1 presents the demographic data from the 2 national surveys conducted in 2013-2014 and 2019. In 2019, the proportions of adolescents who were in a higher school grade (ninth grade), resided in a rural region, and were current cigarette users were somewhat lower than in 2013-2014. Data from 155,117 adolescents (boys: n=82,057, 52.9%) in the 2013-2014 survey and 147,270 adolescents (boys: n=78,789, 53.5%) in the 2019 survey were included in this study.

**Table 1:** Demographics of 2 national surveys conducted in 2013-2014 (n=155,117) and 2019 (n=147,270).

			Prevalence in 2013-2014, %		95% CI		Prevalence in 2019, %		95% CI
**Grade**									
	Seventh	33.1		32.5-33.7		34.8		34.2-35.3	
	Eighth	33.3		32.9-33.7		33.5		33.1-33.9	
	Ninth	33.6		32.9-34.4		31.7		31.2-32.3	
**Sex**									
	Boys	52.9		52.3-53.5		53.5		53.1-54	
	Girls	47.1		46.5-47.7		46.5		46-46.9	
**Residence**									
	Urban	27.9		26-29.8		36.3		34.1-38.5	
	Rural	72.1		70.2-74		63.7		61.5-65.9	
**Region**									
	North	10.6		9.4-11.8		11.1		9.8-12.4	
	East	26.3		24.7-27.9		28.4		26.3-30.6	
	Central	17.1		15.8-18.4		18.3		15.9-20.6	
	South	14.6		12.5-16.8		13.3		11.9-14.8	
	Southwest	16.9		15.3-18.6		16		14.2-17.9	
	Northwest	8.3		7.3-9.4		7.3		6.5-8.1	
	Northeast	6.1		5.1-7.2		5.5		5-6.1	
**Gross domestic product per capita category**									
	Low	27.5		25.5-29.5		27		25.1-28.9	
	Middle	38.4		36.4-40.3		38.6		36.2-41.1	
	High	34.2		32-36.3		34.4		32.1-36.6	
**Cigarette smoking**									
	No	94.1		93.5-94.6		96.1		95.6-96.6	
	Yes	5.9		5.4-6.5		3.9		3.4-4.4	

### Trends in Prevalence of Secondhand Smoke Exposure

**Figure 1:**
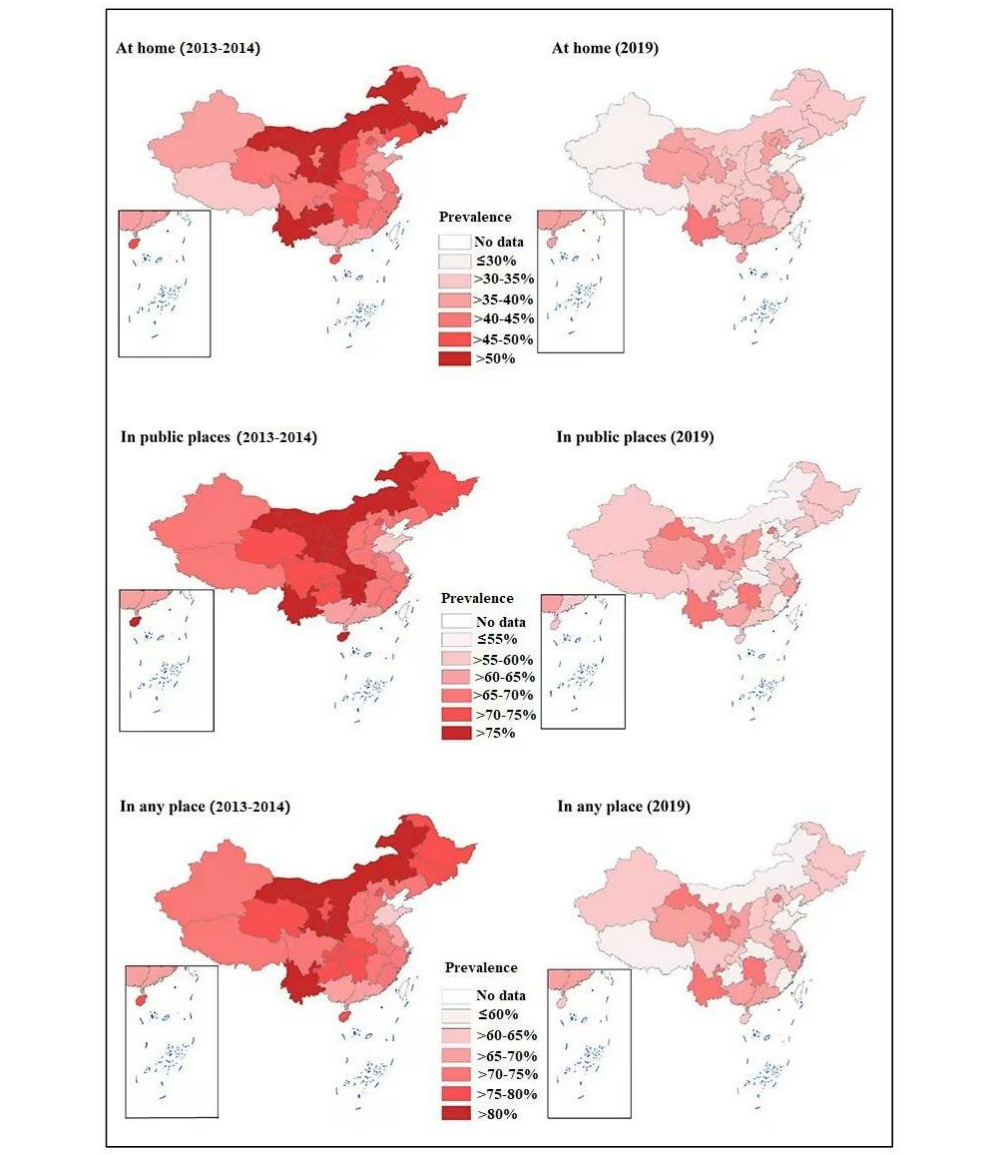
Trends in the geographical distribution of secondhand smoke exposure (on ≥1 day during the past 7 days) at home, in public places, and in any place among Chinese adolescents from 2013-2014 to 2019.

The overall prevalence of secondhand smoke exposure in any place (on ≥1 day during the past 7 days) decreased from 2013-2014 (72.9%, 95% CI 71.5%-74.3%) to 2019 (63.2%, 95% CI 62%-64.5%), while the prevalence of secondhand smoke exposure at home decreased from 44.4% (95% CI 43.1%-45.7%) in 2013-2014 to 34.1% (95% CI 33.1%-35.2%) in 2019, as did secondhand smoke exposure in public places (2013-2014: 68.3%, 95% CI 66.9%-69.6%; 2019: 57.3%, 95% CI 56%-58.6%), as shown in Tables 2, 3, and 4. The prevalence was high in any place, in public places, and at home across all 31 provinces, with the prevalence of secondhand smoke exposure in public places higher compared to exposure at home in each province in both 2013-2014 and 2019 (Figure 1 and Multimedia Appendix 1, Tables S1-S4). The prevalence exceeded 60% in 2019 in 25 of 31 provinces. The 5 provinces where the prevalence was much higher than other provinces were Yunnan (75.1%, 95% CI 66.2%-84%), Hunan (73.6%, 95% CI 68.9%-78.3%), Beijing (73.2%, 95% CI 69.5%-76.9%), Shanghai (72.5%, 95% CI 62.5%-82.5%), and Gansu (70.2%, 95% CI 62.5%-78%). The prevalence of secondhand smoke exposure also decreased according to different exposure frequencies and locations. For example, there was a downward trend in daily exposure in any place (from 31%, 95% CI 29.9%-32.1% in 2013-2014 to 24.7%, 95% CI 23.8%-25.7% in 2019), at home (from 14.2%, 95% CI 13.6%-14.8% in 2013-2014 to 12.4%, 95% CI 11.8%-13% in 2019), and in public places (from 25.1%, 95% CI 24.2%-26% in 2013-2014 to 18.7%, 95% CI 17.8%-19.5% in 2019), as shown in Tables 2, 3, and 4 and Multimedia Appendix 1, Table S5.

The prevalence of secondhand smoke exposure increased as school grade increased regardless of exposure frequency (≥1 day, ≥3 days, ≥5 days, or 7 days during the past 7 days), location (any place, home, or public place), and survey year (2013-2014 or 2019). The prevalence of secondhand smoke exposure decreased as GDP per capita category increased irrespective of exposure frequency, location, or survey year (Tables 2, 3, and 4 and Multimedia Appendix 1, Table S5). The prevalence of exposure was higher among boys (vs girls), current cigarette smokers (vs nonsmokers), and adolescents who lived in an urban region (vs rural region, except for secondhand smoke exposure at home). A higher prevalence of secondhand smoke exposure was observed in the northwest, south, and southwest regions, with lower exposure in the east and northeast regions (Tables 2, 3, and 4). A downward trend in the prevalence of secondhand smoke exposure in any type of public place (closed public places, open public places, and public transportation) and in schools on ≥1 day during the past 7 days was also observed (Multimedia Appendix 1, Tables S6-S7 and Figure S1).

**Table 2:** Trends in the prevalence of secondhand smoke exposure in any place (on ≥1 day during the past 7 days) by age, sex, residence, region, gross domestic product per capita category, and status of cigarette smoking among Chinese adolescents from 2013-2014 to 2019.

		Prevalence in 2013-2014, %	95% CI	Prevalence in 2019, %	95% CI	Absolute change in prevalence, %	95% CI
Overall		72.9	71.5 to 74.3	63.2	62 to 64.5	–9.8	–11.8 to –8.1
**Grade**							
	Seventh	65.7	64 to 67.3	55.8	54 to 57.6	–10	–12.6 to –7.7
	Eighth	74.3	73.5 to 76.2	66.1	64.5 to 67.6	–8.3	–10.9 to –6.2
	Ninth	78.7	77.5 to 79.9	68.5	67.2 to 69.7	–10.3	–12.1 to –8.7
**Sex**							
	Boys	75.5	74.4 to 77	65.4	64 to 66.7	–10.4	–12.3 to –8.7
	Girls	69.9	68.3 to 71.4	60.8	59.4 to 62.2	–9.2	–11.3 to –7.2
**Residence**							
	Urban	74.4	73.3 to 75.6	63.5	61.7 to 65.3	–11	–13.3 to –8.9
	Rural	72.4	70.5 to 74.2	63.1	61.4 to 64.8	–9.4	–12 to –7
**Region**							
	North	73.1	70.3 to 75.9	63	58.8 to 67.3	–10.1	–15.2 to –4.9
	East	68.9	66.8 to 71	59.9	57 to 62.9	–9	–12.6 to –5.4
	Central	75.9	73.9 to 78	64	61.5 to 66.4	–12	–15.2 to –8.8
	South	67.8	61.8 to 73.8	66.3	63.1 to 69.4	–1.7	–8.6 to 5.3
	Southwest	77.5	75.1 to 79.8	65.2	62.1 to 68.2	–12.5	–16.4 to –8.5
	Northwest	78.9	75.4 to 82.3	66.3	63.1 to 69.5	–12.6	–17.3 to –7.8
	Northeast	75.7	73.8 to 77.6	61.2	57.8 to 64.7	–14.5	–18.5 to –10.5
**Gross domestic product per capita category**							
	Low	75.4	73.4 to 77.4	65.4	62.6 to 68.1	–10.1	–13.6 to –6.7
	Middle	75	73.4 to 76.6	64	62.3 to 65.7	–11.1	–13.5 to –8.8
	High	69	66.2 to 71.9	60.7	58.4 to 63.1	–8.3	–12 to –4.6
**Current cigarette smoking**							
	No	71.5	70.1 to 72.9	61.9	60.6 to 63.3	–9.6	–11.1 to –8.1
	Yes	94.1	93.3 to 95	93.8	92.6 to 95	–0.5	–2 to 1.1

**Table 3:** Trends in the prevalence of secondhand smoke exposure at home (on ≥1 day during the past 7 days) by age, sex, residence, region, gross domestic product per capita category, and status of cigarette smoking in Chinese adolescents from 2013-2014 to 2019.

		Prevalence in 2013-2014, %	95% CI		Prevalence in 2019, %		95% CI		Absolute change in prevalence, %		95% CI
Overall		44.4	43.1 to 45.7		34.1		33.1 to 35.2		–10.4		–12 to –8.7
**Grade**											
	Seventh	39.6	38.2 to 40.9	30.5		29.3 to 31.8		–9.1		–11 to –7.3	
	Eighth	46.1	44.4 to 47.7	36		34.7 to 37.3		–10.1		–12.1 to –8.1	
	Ninth	47.5	46.2 to 48.9	36.1		34.9 to 37.3		–11.5		–13.4 to –9.8	
**Sex**											
	Boys	46.4	45.1 to 47.8	36		34.8 to 37.1		–10.5		–12.3 to –8.9	
	Girls	42.1	40.8 to 43.5	32		30.9 to 33.2		–10.2		–12 to –8.4	
**Residence**											
	Urban	43.5	42.1 to 44.8	33.2		31.8 to 34.6		–10.3		–12.3 to –8.3	
	Rural	44.8	43.1 to 46.5	34.6		33.2 to 36.1		–10.2		–12.5 to –8	
**Region**											
	North	46	42.6 to 49.4	35.6		32.1 to 39.2		–10.4		–15.3 to –5.4	
	East	39.6	37.7 to 41.6	31.6		30 to 33.2		–8		–10.6 to –5.5	
	Central	45.8	43.7 to 48	33.7		31.5 to 35.9		–12.1		–15.3 to –9	
	South	40.2	34.9 to 45.5	38		34.1 to 42		–2.2		–8.9 to –4.6	
	Southwest	50.5	47.7 to 53.2	35.7		32.7 to 38.7		–14.8		–19 to –10.7	
	Northwest	48.9	45.7 to 52.1	33.1		29.8 to 36.4		–15.7		–20.4 to –11.1	
	Northeast	46.9	44.6 to 49.2	32.9		30.8 to 35		–14		–17.2 to –10.8	
**Gross domestic product per capita category**											
	Low	48.9	46.9 to 50.9	37		34.4 to 39.5		–11.9		–15.2 to –8.6	
	Middle	44.7	42.9 to 46.4	33.7		32.2 to 35.2		–11		–13.3 to –8.7	
	High	40.8	38.2 to 43.3	32.4		30.8 to 34		–8.4		–11.4 to –5.4	
**Current cigarette smoking**											
	No	42.8	41.5 to 44	32.7		31.7 to 33.7		–10.1		–11.7 to –8.5	
	Yes	68.2	66.6 to 69.7	66.3		64.1 to 68.4		–1.9		–4.6 to 0.7	

**Table 4:** Trends in the prevalence of secondhand smoke exposure in public places (on ≥1 day during the past 7 days) by age, sex, residence, region, gross domestic product per capita category, and status of cigarette smoking among Chinese adolescents from 2013-2014 to 2019.

		Prevalence in 2013-2014, %	95% CI		Prevalence in 2019, %		95% CI		Absolute change in prevalence, %		95% CI
Overall		68.3	66.9 to 69.6		57.3		56 to 58.6		–11		–12.9 to –9.1
**Grade**											
	Seventh	60.2	58.6 to 61.8	48.9		47.1 to 50.6		–11.4		–13.8 to –9	
	Eighth	69.8	68 to 71.5	60.1		58.4 to 61.7		–9.7		–12.2 to –7.3	
	Ninth	74.7	73.5 to 75.9	63.6		62.3 to 65		–11.1		–13 to –9.3	
**Sex**											
	Boys	71.3	70 to 72.5	59.5		58.1 to 60.9		–11.9		–13.7 to –10	
	Girls	64.9	63.4 to 66.4	54.8		53.4 to 56.2		–10.1		–12.2 to –8.1	
**Residence**											
	Urban	69.8	68.6 to 71	57.6		55.8 to 59.5		–12.2		–14.5 to –9.9	
	Rural	67.6	65.9 to 69.4	57.1		55.3 to 58.9		–10.6		–13.1 to –8.1	
**Region**											
	North	67.6	64.7 to 70.5	56.6		52.4 to 60.9		–11		–16.2 to –5.9	
	East	63.5	61.5 to 65.6	53.7		50.7 to 56.6		–9.9		–13.6 to –6.3	
	Central	71.5	69.2 to 73.7	58.5		56 to 61		–13		–16.4 to –9.6	
	South	62.8	57 to 68.6	58.7		55.8 to 61.6		–4.1		–10.7 to 2.5	
	Southwest	73.4	70.8 to 75.9	60.2		57 to 63.4		–13.3		–17.4 to –9.1	
	Northwest	75.1	71.8 to 78.3	61.9		58.1 to 65.6		–13.2		–18.3 to –8.2	
	Northeast	70.3	68.2 to 72.5	55.4		52 to 58.8		–14.9		–19.1 to –10.8	
**Gross domestic product** **per capita category**											
	Low	70.5	68.3 to 72.7	59.5		56.8 to 62.3		–11.1		–14.7 to –7.5	
	Middle	70.7	69.1 to 72.3	58.4		56.6 to 60.2		–12.4		–14.8 to –9.9	
	High	63.7	61 to 66.4	54.3		51.9 to 56.7		–9.4		–13.1 to –5.8	
**Current cigarette smoking**											
	No	66.5	65.2 to 67.9	55.9		54.6 to 57.2		–10.7		–12.6 to –8.8	
	Yes	91.6	90.4 to 92.8	89.9		88.2 to 91.5		–1.9		–3.9 to 0.2	

### Association Between Secondhand Smoke Exposure and Potential Factors

In the multivariable logistic regression analysis, adolescents were more likely to be exposed to secondhand smoke in the combined data set (2013-2014 and 2019) if they were in a higher school grade (ninth vs seventh grade: odds ratio [OR] 1.76, 95% CI 1.68-1.84), were boys (boys vs girls: OR 1.18, 95% CI 1.15-1.22), lived in an urban setting (urban vs rural: OR 1.10, 95% CI 1.01-1.19), and were a current cigarette smoker (smoker vs nonsmoker: OR 6.67, 95% CI 5.83-7.62). These results were largely independent of survey year (2013-2014 or 2019; Table 5).

**Table 5:** Multivariable odds ratios and 95% CIs for factors associated with secondhand smoke exposure (on ≥1 day during the past 7 days) among Chinese adolescents. All variables listed in the table were introduced into logistic regression models.

			In any place, odds ratio (95% CI)							At home, odds ratio (95% CI)							In public places, odds ratio (95% CI)				
			2013-2014		2019		Total		2013-2014			2019		Total		2013-2014			2019		Total
**Grade**																					
	Seventh	1.00		1.00		1.00		1.00			1.00		1.00		1.00			1.00		1.00	
	Eighth	1.49 (1.40-1.60)		1.52 (1.42-1.62)		1.50 (1.44-1.58)		1.28 (1.23-1.34)			1.26 (1.18-1.33)		1.27 (1.22-1.32)		1.50 (1.40-1.59)			1.55 (1.45-1.65)		1.52 (1.45-1.59)	
	Ninth	1.86 (1.75-1.99)		1.66 (1.56-1.78)		1.76 (1.68-1.84)		1.34 (1.28-1.40)			1.23 (1.17-1.30)		1.29 (1.25-1.34)		1.89 (1.77-2.01)			1.77 (1.65-1.90)		1.83 (1.74-1.91)	
**Sex**																					
	Girls	1.00		1.00		1.00		1.00			1.00		1.00		1.00			1.00		1.00	
	Boys	1.23 (1.19-1.30)		1.16 (1.12-1.20)		1.18 (1.15-1.22)		1.10 (1.06-1.14)			1.12 (1.09-1.16)		1.10 (1.08-1.12)		1.25 (1.20-1.30)			1.16 (1.11-1.20)		1.18 (1.11-1.20)	
**Residence**																					
	Rural	1.00		1.00		1.00		1.00			1.00		1.00		1.00			1.00		1.00	
	Urban	1.22 (1.09-1.37)		1.09 (0.98-1.22)		1.10 (1.01-1.19)		1.01 (0.92-1.10)			0.99 (0.91-1.08)		0.96 (0.89-1.03)		1.22 (1.10-1.35)			1.11 (0.99-1.23)		1.10 (1.02-1.19)	
**Region**																					
	North	1.00		1.00		1.00		1.00			1.00		1.00		1.00			1.00		1.00	
	East	0.91 (0.73-1.14)		0.96 (0.77-1.21)		0.93 (0.79-1.09)		0.86 (0.71-1.03)			0.93 (0.77-1.12)		0.89 (0.77-1.01)		0.92 (0.75-1.13)			0.97 (0.78-1.21)		0.94 (0.80-1.10)	
	Central	1.18 (0.95-1.46)		1.06 (0.85-1.32)		1.09 (0.93-1.28)		1.07 (0.9-1.28)			0.95 (0.78-1.16)		1.00 (0.87-1.15)		1.20 (0.98-1.46)			1.11 (0.89-1.37)		1.12 (0.96-1.31)	
	South	0.80 (0.61-1.05)		1.20 (0.95-1.52)		0.99 (0.82-1.19)		0.81 (0.65-1.01)			1.16 (0.92-1.47)		0.97 (0.82-1.14)		0.83 (0.64-1.07)			1.14 (0.92-1.40)		0.98 (0.82-1.16)	
	Southwest	1.24 (1.01-1.51)		1.06 (0.84-1.34)		1.13 (0.96-1.33)		1.17 (0.98-1.39)			0.97 (0.79-1.18)		1.07 (0.94-1.23)		1.28 (1.06-1.54)			1.12 (0.90-1.41)		1.19 (1.02-1.39)	
	Northwest	1.37 (1.07-1.77)		1.15 (0.90-1.45)		1.26 (1.05-1.51)		1.18 (0.98-1.42)			0.90 (0.73-1.12)		1.06 (0.92-1.23)		1.42 (1.14-1.77)			1.24 (0.97-1.58)		1.34 (1.12-1.59)	
	Northeast	1.13 (0.94-1.34)		0.91 (0.72-1.15)		1.02 (0.87-1.20)		1.05 (0.89-1.25)			0.90 (0.75-1.07)		1.00 (0.88-1.13)		1.10 (0.93-1.30)			0.94 (0.75-1.17)		1.03 (0.89-1.20)	
**Gross domestic product per capita category**																					
	Low	1.00		1.00		1.00		1.00			1.00		1.00		1.00			1.00		1.00	
	Middle	0.96 (0.81-1.14)		0.99 (0.85-1.15)		0.98 (0.87-1.10)		0.87 (0.76-0.99)			0.95 (0.82-1.09)		0.91 (0.82-1.00)		0.99 (0.85-1.16)			0.99 (0.85-1.14)		0.99 (0.89-1.11)	
	High	0.86 (0.69-1.07)		0.87 (0.73-1.03)		0.88 (0.76-1.01)		0.88 (0.75-1.04)			0.88 (0.75-1.03)		0.89 (0.79-1.00)		0.86 (0.70-1.06)			0.87 (0.74-1.02)		0.88 (0.77-1.00)	
**Current cigarette smoking**																					
	No	1.00		1.00		1.00		1.00			1.00		1.00		1.00			1.00		1.00	
	Yes	5.31 (4.45-6.35)		8.07 (6.60-9.87)		6.67 (5.83-7.62)		2.51 (2.31-2.73)			3.65 (3.36-3.96)		3.04 (2.87-3.22)		4.53 (3.89-5.28)			6.10 (5.08-7.32)		5.39 (4.80-6.05)	

### Trends in Attitude Toward Secondhand Smoke Exposure

We observed no change in the proportion of adolescents who thought secondhand smoke exposure was harmful to them between the surveys (2013-2014: 73.9%, 95% CI 73.3%-74.5%; 2019: 73.7%, 95% CI 72.7%-74.8%). This observation was consistent across subgroups by grade, residence, region, and GDP per capita category. However, we noted fewer girls and cigarette smokers and more boys, proportionally, considered smoking to be harmful in 2019 compared to 2013-2014 (Multimedia Appendix 1, Table S8).

## Discussion

### Principal Findings

A decrease in the prevalence of secondhand smoke exposure occurred from 2013-2014 to 2019 among middle school–aged students in China. This decline was observed irrespective of sex, residence, region, provincial economic level, and location of exposure (in any place, at home, and in public places). However, the prevalence remained high in 2019 (63.2% in any place, 34.1% at home, and 57.3% in public places), highlighting the need for more effective strategies to control tobacco use and further curtail secondhand smoke exposure in China.

To the best of our knowledge, this is the first study to assess in detail the latest information on the prevalence of secondhand smoke exposure and trends in this exposure among adolescents in China using nationally representative data. Overall, in 2019 the prevalence (on ≥1 day during the past 7 days) was 34.1% (95% CI 33.1%-35.2%) at home, which is higher than in the United States (25.3%, 95% CI 23.4%-27.3%), while the prevalence of exposure was similar on public transportation between the 2 countries (China: 23.4%, 95% CI 22.3%-24.4%; United States: 23.3%, 95% CI 21.4%-25.4%) [17]. The prevalence of exposure to secondhand smoke was 57.3% (95% CI 56%-58.6%) in public places and 63.2% (95% CI 62%-64.5%) in any place in 2019, which was higher than in many other countries [9,18,19]. In addition, nearly a quarter (24.7%, 95% CI 23.8%-25.7%) of adolescents were exposed to secondhand smoke in any place daily, one-tenth (12.4%, 95% CI 11.8%-13%) at home and one-fifth (18.7%, 95% CI 17.8%-19.5%) in public places. Data from the 2010-2018 GYTS among 711,366 adolescents aged 12 to 16 years in 142 countries showed that the global prevalence of secondhand smoke exposure (on ≥1 day) was 33.1% (95% CI 32.1%-34.1%) at home, 57.6% (95% CI 56.4%-58.8%) in public places, and 62.9% (95% CI 61.7%-64.1%) in any place; the daily exposure prevalence was 12.3% (95% CI 11.7%-13%) at home, 23.5% (95% CI 22.5%-24.5%) in public places, and 32.5% (95% CI 31.5%-33.6%) in any place [9]. While these data highlight the fact that secondhand smoke exposure remains a serious public health issue worldwide, our data, using the same instruments of exposure measurement, show that exposure in China is comparatively much higher.

One advantage of our data over previous studies is that our exposure assessment was able to further categorize the types of public places where exposure occurred. It is obvious that the health risk should be higher for exposure in enclosed public places than open public places. Our findings showed that the prevalence in enclosed public places (45.8%) was similar to that in open public places (48.6%) in 2019, although the prevalence was lower on public transportation (23.4%). Another important finding is that in 2019 nearly 50% of adolescents were exposed to secondhand smoke in schools, suggesting that many school staff are cigarette smokers who not only set a bad example for students, but also expose students to their secondhand smoke. These data reinforce the fact that secondhand smoke exposure among adolescents remains a serious public health issue, especially in public places, and identifies key exposure sites outside of the home that could be targeted for intervention.

We observed that the prevalence of secondhand smoke exposure increased as school grade increased, which can be explained as older adolescents from higher grades being more accepting of tobacco use among their peers and being more likely to smoke themselves; both factors increase the probability of secondhand smoke exposure [14,20]. We also found that the prevalence of exposure decreased when moving from low to high provincial GDP per capita categories, as others have shown [21,22]. This might be partly explained by lower awareness of the harm of secondhand smoke exposure among adolescents in lower GDP per capita regions than higher GDP per capita regions. Moreover, many economically developed municipalities, such as Beijing and Shanghai, have implemented stricter smoke-free policies than other municipalities or provinces. Notably, however, there was little difference observed in the prevalence of secondhand smoke exposure among adolescents across the 7 geographical regions. These findings further emphasize the fact that secondhand smoke exposure among adolescents remains a serious concern across mainland China. We found that almost all adolescent cigarette smokers (89.9%) were exposed to secondhand smoke (≥1 day during the past 7 days), although exposure was also high in adolescents who did not smoke cigarettes (55.9%). These data are consistent with those from other studies showing a positive relationship between secondhand smoke exposure and cigarette smoking [20,23]. This finding further suggests that preventing smoking among adolescents would help lower the prevalence of secondhand smoke exposure among this population.

Of note, the prevalence of secondhand smoke exposure among adolescents decreased by approximately 10% from 2013-2014 to 2019 regardless of location (in any place, at home, and in public places). In 2005, China ratified the WHO Framework Convention on Tobacco Control (FCTC), which came into force on January 9, 2006 [24]. Since then, a series of tobacco control measures have been drafted in China. In 2007, the Inter-Ministry Coordination and Steering Committee for Implementation of WHO FCTC was established under the State Council [25]. In 2009, the Ministry of Finance and State Taxation Administration decided to adjust the taxation of tobacco products. In 2000 (when the exchange rate was US $1=RMB 8.28), the tax rate was 45% for tobacco products priced RMB ≥50/carton and 30% for products priced RMB <50/carton; in 2009 (when the exchange rate was US $1=RMB 6.81), the rates were 56% for products priced RMB ≥70/carton and 36% for products priced RMB <70/carton) [26]. To protect youth from secondhand smoke exposure, the General Office of the Ministry of Education further strengthened tobacco control in schools (ie, strengthening tobacco control propaganda and education, ensuring that teachers set a good example for students, and establishing a perfect system for tobacco control) in 2010 [27]. Based on the actions above, China has made some progress in tobacco control. It was reported that the prevalence of secondhand smoke exposure among mainly adult people aged ≥15 years declined from 55.2% (95% CI 50.4%-59.9%) in 2010 to 45.3% (95% CI 41.4%-49.2%) in 2015 [7]. Despite these efforts, tobacco control is only slowly improving in China. We found that the prevalence of secondhand smoke exposure at home and in public places is still unacceptably high. To investigate whether a hygiene intervention for Chinese smokers in households with young children would lead to a reduction of secondhand smoke exposure among children, 180 families were randomly divided into an intervention group (n=98 families) and a control group (n=82 families). The results suggested that a smoking hygiene intervention might be an effective measure in reducing the prevalence of secondhand smoke exposure among children [28]. Smoke-free car laws have also been tested as rational and effective measures in reducing secondhand smoke exposure among youth [29,30]. Furthermore, we found that nearly three-fourths of adolescents are aware of the harms to them of secondhand smoke. Feng et al [31] reported that 68.5% of adult nonsmokers and 60.6% of adult smokers believed that secondhand smoke exposure could cause lung cancer. This provides a solid foundation for national smoke-free legislation. These findings call for stronger action to promote the process of achieving a smoke-free world.

### Limitations

Although our study has several strengths (2 surveys were conducted in a nationally representative sample of Chinese adolescents and followed strict quality assurance and control procedures to ensure the validity and reliability of the data; to our knowledge, this study reports the most recent prevalence and trend estimates of secondhand smoke exposure among adolescents in mainland China), several limitations should be noted. First, the prevalence of secondhand smoke exposure was estimated by self-report, which is subject to recall bias. However, the questions used to collect the exposure data have been tested with good reliability and adequate validity [32,33]. Further studies using objective biomarkers of secondhand smoke exposure (eg, plasma, saliva, or urinary cotinine or other nicotine biomarkers) could improve the reliability of our results. Second, each of the 2 surveys was a school-based survey, and our estimates of prevalence are conservative given that risk behaviors among those who leave school may be higher than among those who remain in school [34]. Third, only middle school students were included in our analyses; thus, our findings could not be directly generalized to youth in other age groups. Fourth, our study did not provide enough information on secondhand smoke exposure (eg, exposure to different types of tobacco and exposure duration per day). Fifth, data from Taiwan, Hong Kong, and Macao were missing, thus limiting the generalizability of our findings to adolescents throughout China. Sixth, a causal association should not necessarily be inferred, because this study had a cross-sectional design. Seventh, many other factors influencing secondhand smoke exposure, such as social, economic, and individual factors, were not considered in this study.

### Conclusions

Although the prevalence of secondhand smoke exposure among Chinese adolescents has declined from 2013-2014 to 2019, exposure remains unacceptably high. These findings suggest more effective strategies and stronger action, especially in public places, but also at home, are needed in China to further curtail secondhand smoke exposure among adolescents.

## Appendix

Multimedia Appendix 1 Supplementary files.

## Abbreviations

FCTC: Framework Convention on Tobacco Control
GDP: gross domestic product
GYTS: Global Youth Tobacco Survey
NYTS: China National Youth Tobacco Survey
OR: odds ratio
WHO: World Health Organization

## Data Availability

The data sets generated during and/or analyzed during the current study are available from the corresponding author S Liu on reasonable request.
