# Supplementary material for: Trends in Exposure to Secondhand Smoke Among Adolescents in China From 2013-2014 to 2019: Two Repeated National Cross-sectional Surveys
Source: JMIR Public Health Surveill. 2023 Mar 24;9:e40782. doi: 10.2196/40782 (PMC10131728; doi:10.2196/40782)
Supplement: Multimedia Appendix 1 [file publichealth_v9i1e40782_app1.doc]

**Multimedia Appendix 1: Supplementary files.
Table S1. Prevalence of secondhand smoke exposure (on ≥1 day during the past 7 days) by province in Chinese youth from 2013-14 to 2019**

| Province | In any place | |  | At home | |  | In public places | |
| --- | --- | --- | --- | --- | --- | --- | --- | --- |
|  | 2013-14 | 2019 |  | 2013-14 | 2019 |  | 2013-14 | 2019 |
| Yunnan | 82.1 (76.7,87.4) | 75.1 (66.2,84.0) |  | 60.4 (55.3,65.5) | 43.8 (33.5,54.0) |  | 78.5 (72.6,84.4) | 70.1 (60.7,79.5) |
| Neimenggu | 81.7 (77.1,86.4) | 58.9 (57.0,60.7) |  | 55.1 (49.7,60.5) | 34.0 (31.5,36.5) |  | 77.4 (73.0,81.7) | 52.5 (50.6,54.4) |
| Gansu | 81.6 (79.0,84.2) | 70.2 (62.5,78.0) |  | 55.5 (53.1,57.8) | 38.7 (30.0,47.3) |  | 77.5 (75.1,79.9) | 65.3 (56.2,74.5) |
| Shaanxi | 81.6 (77.4,85.7) | 65.9 (60.8,71.0) |  | 51.9 (49.7,56.0) | 33.4 (27.5,39.3) |  | 78.2 (74.1,82.3) | 61.7 (56.8,66.6) |
| Hunan | 80.1 (76.5,83.8) | 73.6 (68.9,78.3) |  | 49.2 (43.7,54.7) | 38.2 (31.2,45.2) |  | 76.3 (72.8,79.8) | 69.7 (66.1,73.2) |
| Hubei | 79.9 (76.4,83.3) | 58.2 (50.0,66.5) |  | 48.8 (44.8,52.8) | 32.0 (26.1,38.0) |  | 76.0 (72.0,80.0) | 52.6 (44.7,60.5) |
| Hainan | 79.4 (75.1,83.7) | 64.6 (60.1,69.0) |  | 49.1 (43.7,54.6) | 39.0 (35.7,42.2) |  | 75.6 (71.1,80.2) | 58.5 (54.0,63.1) |
| Ningxia | 79.2 (73.4,85.0) | 67.1 (63.4,70.7) |  | 43.8 (39.1,48.5) | 30.4 (27.1,33.7) |  | 76.4 (70.1,82.6) | 63.3 (59.5,67.0) |
| Guizhou | 78.6 (75.4,81.7) | 59.7 (53.3,66.1) |  | 53.8 (50.5,57.1) | 34.3 (29.3,39.3) |  | 73.9 (69.3,78.6) | 54.5 (48.4,60.5) |
| Beijing | 78.0 (74.8,81.3) | 73.2 (69.5,76.9) |  | 46.4 (40.6,52.2) | 35.7 (31.0,40.5) |  | 72.9 (69.6,76.1) | 67.2 (61.8,72.7) |
| Jilin | 78.4 (75.1,81.6) | 61.0 (53.3,68.6) |  | 51.8 (48.2,55.5) | 32.3 (29.6,35.0) |  | 72.0 (67.8,76.2) | 55.5 (47.0,64.0) |
| Qinghai | 77.5 (61.4,93.6) | 67.6 (60.5,74.7) |  | 43.2 (35.2,51.2) | 35.9 (28.9,42.9) |  | 74.6 (57.5,91.7) | 63.0 (56.1,69.8) |
| Heilongjiang | 74.8 (71.0,78.5) | 60.7 (55.8,65.7) |  | 44.7 (40.1,49.4) | 33.5 (29.8,37.1) |  | 70.1 (66.4,73.7) | 55.5 (51.0,60.1) |
| Chongqing | 75.1 (69.3,80.9) | 62.0 (58.3,65.7) |  | 43.5 (37.3,49.6) | 30.2 (28.1,32.2) |  | 71.6 (65.9,77.3) | 56.9 (52.5,61.3) |
| Liaoning | 74.4 (71.5,77.2) | 61.9 (56.2,67.6) |  | 46.0 (43.6,48.4) | 32.8 (29.0,36.6) |  | 69.6 (66.1,73.0) | 55.3 (50.0,60.6) |
| Sichuan | 74.5 (70.0,79.0) | 63.4 (60.8,66.1) |  | 44.4 (38.4,50.3) | 33.7 (29.5,37.9) |  | 70.2 (65.7,74.6) | 58.6 (55.8,61.3) |
| Jiangxi | 73.3 (70.8,75.8) | 60.6 (53.3,68.0) |  | 40.8 (36.0,45.6) | 30.7 (26.4,35.0) |  | 69.3 (66.7,71.8) | 55.5 (48.1,62.9) |
| Shanghai | 73.0 (69.5,76.6) | 72.5 (62.5,82.5) |  | 41.5 (37.2,45.8) | 37.8 (32.7,42.9) |  | 67.1 (63.6,70.6) | 65.3 (53.8,76.8) |
| Zhejiang | 72.9 (67.8,77.9) | 66.1 (62.4,69.7) |  | 43.1 (36.8,49.5) | 33.6 (30.4,36.9) |  | 67.3 (61.8,72.9) | 60.3 (56.1,64.4) |
| Fujian | 72.6 (71.1,74.2) | 58.5 (52.7,64.2) |  | 43.9 (39.8,47.9) | 30.7 (28.1,33.3) |  | 66.8 (65.3,68.3) | 52.9 (46.5,59.4) |
| Henan | 72.4 (69.3,75.4) | 60.8 (58.5,63.1) |  | 43.0 (40.4,45.7) | 31.9 (30.2,33.6) |  | 67.5 (64.1,71.0) | 54.6 (52.3,56.9) |
| Hebei | 71.7 (68.1,75.2) | 62.3 (55.1,69.5) |  | 43.6 (37.7,49.4) | 36.5 (31.1,41.9) |  | 65.7 (62.0,69.3) | 54.8 (47.6,62.0) |
| Xinjiang | 68.8 (50.3,87.4) | 62.7 (55.9,69.4) |  | 37.6 (21.5,53.7) | 27.8 (22.8,32.8) |  | 67.2 (53.3,81.2) | 58.1 (49.1,67.0) |
| Xizang | 71.5 (65.8,77.2) | 59.2 (49.5,68.9) |  | 32.9 (28.3,37.5) | 22.9 (18.9,26.9) |  | 68.7 (61.5,75.8) | 56.7 (46.1,67.4) |
| Shanxi | 71.3 (64.6,77.9) | 65.0 (58.0,72.1) |  | 45.6 (39.6,51.6) | 34.6 (26.2,43.0) |  | 65.8 (58.9,72.7) | 60.9 (54.7,67.1) |
| Anhui | 70.7 (63.9,77.6) | 65.3 (57.4,73.3) |  | 39.1 (32.8,45.3) | 38.1 (31.9,44.3) |  | 66.6 (60.0,73.1) | 57.2 (48.5,66.0) |
| Guangxi | 68.3 (60.4,76.3) | 67.5 (61.9,73.2) |  | 39.8 (34.8,44.8) | 38.5 (31.5,45.5) |  | 63.2 (54.9,71.6) | 60.5 (55.9,65.0) |
| Jiangsu | 68.1 (61.9,74.2) | 63.2 (61.0,65.3) |  | 40.0 (35.1,45.0) | 33.9 (32.0,35.9) |  | 62.8 (56.5,69.1) | 56.5 (54.1,58.9) |
| Guangdong | 65.9 (56.9,74.9) | 65.7 (61.5,69.9) |  | 39.1 (31.0,47.2) | 37.7 (32.5,42.9) |  | 61.1 (53.2,69.1) | 57.8 (53.7,61.8) |
| Tianjin | 64.1 (54.5,73.6) | 62.4 (59.0,65.9) |  | 40.7 (37.7,43.6) | 34.5 (29.1,39.9) |  | 58.1 (49.3,66.9) | 55.9 (51.7,60.1) |
| Shandong | 62.5 (58.0,67.0) | 50.3 (42.3,58.3) |  | 35.9 (32.7,39.1) | 25.2 (22.4,28.1) |  | 56.4 (52.4,60.4) | 44.3 (36.1,52.4) |

Data are presented as %(95%CI).

**Table S2. Prevalence of secondhand smoke exposure (on ≥3 days during the past 7 days) by province in Chinese youth from 2013-14 to 2019**

| Province | In any place | |  | At home | |  | In public places | |
| --- | --- | --- | --- | --- | --- | --- | --- | --- |
|  | 2013-14 | 2019 |  | 2013-14 | 2019 |  | 2013-14 | 2019 |
| Yunnan | 64.1 (56.8,71.4) | 50.7 (42.6,58.8) |  | 36.0 (30.9,41.1) | 21.5 (13.8,29.1) |  | 57.4 (49.7,65.1) | 45.5 (37.5,53.4) |
| Neimenggu | 60.6 (52.8,68.4) | 40.4 (38.4,42.5) |  | 32.6 (26.1,39.1) | 22.3 (20.8,23.8) |  | 53.1 (45.4,60.8) | 33.2 (30.1,36.3) |
| Gansu | 60.4 (57.5,63.4) | 49.5 (39.9,59.0) |  | 30.9 (27.3,34.5) | 21.4 (15.6,27.2) |  | 54.2 (51.1,57.3) | 44.7 (35.0,54.4) |
| Shaanxi | 59.3 (52.7,66.0) | 44.9 (39.4,50.4) |  | 26.2 (21.8,30.7) | 18.6 (14.4,22.8) |  | 54.7 (48.2,61.2) | 40.0 (35.1,44.9) |
| Hunan | 57.7 (53.8,61.7) | 55.4 (52.3,58.5) |  | 25.5 (22.5,28.5) | 23.9 (21.7,26.1) |  | 52.3 (48.5,56.2) | 50.3 (47.4,53.1) |
| Hubei | 57.5 (51.5,63.5) | 36.3 (30.3,42.3) |  | 24.8 (22.0,27.6) | 17.6 (13.5,21.7) |  | 52.5 (46.0,59.0) | 31.4 (26.1,36.8) |
| Hainan | 61.5 (56.2,66.8) | 46.5 (41.3,51.7) |  | 30.0 (25.1,34.9) | 24.8 (21.9,27.7) |  | 55.8 (50.3,61.3) | 39.9 (35.0,44.8) |
| Ningxia | 56.2 (49.9,62.6) | 45.6 (41.0,50.2) |  | 24.5 (20.4,28.5) | 16.4 (13.8,19.0) |  | 50.5 (43.9,57.0) | 40.9 (36.7,45.0) |
| Guizhou | 56.0 (51.3,60.7) | 37.4 (32.0,42.8) |  | 28.0 (24.2,31.8) | 18.3 (15.3,21.3) |  | 50.4 (46.0,54.9) | 31.3 (26.2,36.3) |
| Beijing | 56.2 (51.5,60.8) | 50.7 (47.8,53.6) |  | 28.7 (25.1,32.3) | 23.9 (21.0,26.8) |  | 49.1 (45.2,52.9) | 43.6 (39.5,47.7) |
| Jilin | 56.2 (51.8,60.5) | 42.7 (37.3,48.1) |  | 32.3 (28.7,36.0) | 21.6 (19.2,24.0) |  | 46.4 (41.1,51.7) | 36.5 (31.3,41.7) |
| Qinghai | 58.6 (42.9,74.4) | 49.3 (43.0,55.7) |  | 26.1 (18.6,33.6) | 21.9 (17.8,26.0) |  | 53.3 (38.1,68.6) | 43.2 (36.5,49.9) |
| Heilongjiang | 55.0 (52.1,58.0) | 43.0 (37.9,48.2) |  | 29.2 (26.2,32.2) | 23.2 (20.9,25.5) |  | 47.5 (44.1,50.9) | 36.1 (31.0,41.2) |
| Chongqing | 53.7 (47.5,60.0) | 40.3 (37.2,43.4) |  | 23.5 (19.5,27.4) | 17.9 (16.8,19.1) |  | 49.2 (42.9,55.5) | 34.7 (31.4,38.1) |
| Liaoning | 54.2 (50.7,57.7) | 44.7 (39.3,50.1) |  | 29.0 (27.7,30.4) | 23.1 (19.2,27.0) |  | 46.8 (42.5,51.1) | 37.3 (32.3,42.2) |
| Sichuan | 52.7 (47.4,58.0) | 40.8 (38.2,43.5) |  | 25.3 (20.4,30.2) | 17.9 (15.2,20.6) |  | 47.2 (42.8,51.5) | 35.6 (33.2,38.1) |
| Jiangxi | 53.8 (50.6,57.0) | 42.8 (36.1,49.4) |  | 23.0 (18.8,27.1) | 17.9 (15.2,20.5) |  | 48.4 (45.7,51.1) | 37.3 (30.8,43.8) |
| Shanghai | 50.2 (45.8,54.6) | 48.3 (39.9,56.8) |  | 26.5 (23.1,29.9) | 26.4 (22.9,29.9) |  | 40.9 (36.6,45.2) | 37.5 (28.5,46.6) |
| Zhejiang | 47.1 (41.8,52.4) | 42.3 (37.5,47.2) |  | 22.2 (18.1,26.3) | 19.6 (16.9,22.3) |  | 41.3 (36.0,46.5) | 36.2 (31.5,40.9) |
| Fujian | 51.3 (49.5,53.2) | 39.0 (33.0,45.0) |  | 26.2 (23.0,29.5) | 19.0 (16.3,21.8) |  | 44.3 (42.6,46.1) | 32.8 (26.6,39.1) |
| Henan | 48.1 (44.2,52.1) | 37.4 (35.4,39.3) |  | 21.3 (18.3,24.4) | 16.7 (15.7,17.8) |  | 42.6 (39.2,46.0) | 31.7 (29.8,33.5) |
| Hebei | 49.6 (45.3,53.9) | 42.1 (35.7,48.6) |  | 23.4 (19.9,26.9) | 21.9 (17.8,26.1) |  | 42.5 (38.1,46.9) | 34.2 (28.4,40.0) |
| Xinjiang | 49.2 (35.4,62.9) | 37.2 (30.5,44.0) |  | 21.7 (11.9,31.6) | 15.2 (11.5,18.8) |  | 43.6 (31.0,56.2) | 32.2 (25.6,38.9) |
| Xizang | 46.4 (37.9,54.8) | 35.9 (25.3,46.6) |  | 11.5 (10.0,13.0) | 9.1 (6.6,11.7) |  | 43.6 (34.9,52.3) | 33.4 (23.0,43.8) |
| Shanxi | 50.9 (45.4,56.4) | 46.0 (40.2,51.9) |  | 24.3 (22.1,26.6) | 19.4 (14.7,24.1) |  | 44.3 (38.1,50.4) | 41.7 (36.4,46.9) |
| Anhui | 46.8 (40.6,53.0) | 41.8 (34.6,48.9) |  | 19.7 (15.7,23.8) | 21.4 (17.4,25.5) |  | 42.0 (36.3,47.6) | 33.7 (26.9,40.5) |
| Guangxi | 46.9 (40.1,53.7) | 43.0 (38.2,47.8) |  | 21.4 (18.5,24.3) | 21.1 (16.4,25.9) |  | 42.0 (34.7,49.4) | 35.5 (32.5,38.5) |
| Jiangsu | 44.7 (38.1,51.3) | 38.7 (36.6,40.8) |  | 21.7 (17.4,26.0) | 20.0 (17.9,22.0) |  | 38.1 (32.3,44.0) | 30.9 (29.1,32.7) |
| Guangdong | 48.0 (39.7,56.2) | 43.4 (39.1,47.6) |  | 23.4 (17.8,29.1) | 23.1 (18.9,27.3) |  | 41.2 (34.4,48.0) | 34.4 (30.6,38.2) |
| Tianjin | 46.8 (37.7,55.9) | 46.1 (41.9,50.4) |  | 26.6 (22.7,30.5) | 24.7 (20.4,29.1) |  | 39.5 (30.9,48.1) | 38.3 (34.0,42.5) |
| Shandong | 36.8 (33.7,40.0) | 30.4 (23.8,36.9) |  | 17.2 (15.3,19.1) | 14.7 (11.6,17.8) |  | 30.5 (27.6,33.4) | 24.7 (18.5,30.9) |

Data are presented as %(95%CI).

**Table S3. Prevalence of secondhand smoke exposure (on ≥5 days during the past 7 days) by province in Chinese youth from 2013-14 to 2019**

| Province | In any place | |  | At home | |  | In public places | |
| --- | --- | --- | --- | --- | --- | --- | --- | --- |
|  | 2013-14 | 2019 |  | 2013-14 | 2019 |  | 2013-14 | 2019 |
| Yunnan | 50.6 (43.2,57.9) | 36.6 (29.4,43.7) |  | 26.1 (22.7,29.6) | 14.7 (8.9,20.5) |  | 43.2 (35.6,50.8) | 31.4 (25.1,37.6) |
| Neimenggu | 47.5 (38.9,56.2) | 30.2 (27.9,32.4) |  | 25.1 (18.9,31.3) | 16.5 (15.3,17.8) |  | 38.8 (31.0,46.6) | 22.8 (19.9,25.7) |
| Gansu | 46.8 (43.6,49.9) | 37.9 (30.5,45.2) |  | 21.7 (18.8,24.6) | 16.4 (12.2,20.6) |  | 39.9 (36.7,43.1) | 32.4 (25.2,39.5) |
| Shaanxi | 43.9 (37.5,50.3) | 32.3 (27.7,36.9) |  | 17.3 (13.7,20.8) | 13.4 (10.8,15.9) |  | 39.2 (33.2,45.2) | 27.3 (22.9,31.6) |
| Hunan | 42.4 (38.9,45.8) | 41.4 (39.3,43.6) |  | 17.6 (15.6,19.7) | 18.2 (17.1,19.2) |  | 37.0 (34.0,40.0) | 35.0 (31.2,38.8) |
| Hubei | 43.7 (37.6,49.7) | 26.3 (21.8,30.7) |  | 17.6 (15.1,20.1) | 12.8 (9.8,15.7) |  | 37.9 (32.1,43.7) | 21.5 (18.0,25.0) |
| Hainan | 49.1 (44.5,53.7) | 35.9 (31.7,40.1) |  | 22.6 (18.6,26.7) | 18.9 (16.8,21.0) |  | 42.8 (38.7,46.8) | 29.0 (24.9,33.0) |
| Ningxia | 41.6 (35.4,47.9) | 32.0 (28.1,35.9) |  | 16.7 (13.6,19.8) | 11.9 (9.9,13.9) |  | 35.2 (29.1,41.3) | 26.8 (23.1,30.5) |
| Guizhou | 41.4 (36.7,46.1) | 26.8 (23.0,30.7) |  | 19.6 (16.6,22.6) | 13.4 (10.8,15.9) |  | 35.4 (30.8,39.9) | 20.8 (16.8,24.8) |
| Beijing | 43.4 (39.5,47.2) | 36.7 (34.6,38.9) |  | 21.1 (18.5,23.7) | 18.2 (15.9,20.5) |  | 35.8 (33.0,38.6) | 29.3 (26.5,32.1) |
| Jilin | 43.3 (39.4,47.1) | 33.0 (29.3,36.7) |  | 24.1 (20.6,27.5) | 17.3 (15.6,19.0) |  | 32.7 (28.6,36.7) | 26.2 (22.5,30.0) |
| Qinghai | 44.6 (31.8,57.5) | 34.8 (29.3,40.3) |  | 19.1 (14.3,23.8) | 15.1 (11.7,18.5) |  | 38.6 (26.4,50.8) | 28.6 (23.7,33.5) |
| Heilongjiang | 42.5 (39.8,45.1) | 32.9 (28.9,37.0) |  | 22.1 (19.4,24.7) | 18.2 (16.1,20.3) |  | 34.3 (31.9,36.7) | 25.8 (22.1,29.4) |
| Chongqing | 40.9 (35.0,46.8) | 29.5 (27.2,31.8) |  | 16.9 (13.5,20.3) | 13.6 (12.5,14.7) |  | 35.7 (29.9,41.5) | 23.8 (21.8,25.8) |
| Liaoning | 41.6 (37.5,45.8) | 34.9 (29.9,39.8) |  | 22.1 (20.7,23.5) | 18.3 (14.8,21.8) |  | 33.3 (28.7,38.0) | 26.9 (22.9,31.0) |
| Sichuan | 40.2 (34.5,46.0) | 29.6 (27.4,31.8) |  | 18.8 (14.8,22.8) | 13.1 (10.5,15.6) |  | 34.2 (29.3,39.0) | 24.3 (22.3,26.3) |
| Jiangxi | 40.0 (37.2,42.8) | 31.5 (24.6,38.3) |  | 15.6 (12.6,18.5) | 13.2 (10.5,15.8) |  | 34.7 (32.0,37.5) | 25.8 (19.7,31.9) |
| Shanghai | 37.4 (33.1,41.7) | 33.8 (28.3,39.4) |  | 19.8 (17.6,22.1) | 19.4 (17,21.9) |  | 27.7 (24.0,31.5) | 22.8 (17.5,28.2) |
| Zhejiang | 35.3 (31.3,39.4) | 29.5 (24.0,34.9) |  | 16.0 (13.4,18.6) | 15.4 (13.1,17.7) |  | 29.2 (25.2,33.2) | 22.8 (17.7,27.8) |
| Fujian | 38.0 (35.7,40.3) | 29.9 (25.1,34.6) |  | 18.9 (16.5,21.4) | 15.1 (13.0,17.3) |  | 30.9 (29.1,32.7) | 23.6 (18.6,28.6) |
| Henan | 34.6 (31.3,37.9) | 25.9 (24.1,27.6) |  | 14.6 (12.3,17.0) | 11.5 (10.6,12.4) |  | 29.2 (26.4,32.0) | 20.8 (19.1,22.5) |
| Hebei | 35.7 (31.3,40.1) | 30.5 (24.8,36.3) |  | 16.0 (13.5,18.5) | 15.9 (12.6,19.2) |  | 29.0 (24.6,33.5) | 23.1 (18.3,27.9) |
| Xinjiang | 34.2 (23.2,45.2) | 24.0 (18.8,29.3) |  | 15.1 (7.7,22.4) | 10.4 (7.5,13.4) |  | 27.7 (19.5,35.8) | 19.1 (14.1,24.1) |
| Xizang | 31.0 (24.8,37.3) | 22.4 (14.1,30.8) |  | 7.3 (5.9,8.8) | 6.2 (3.4,9.0) |  | 28.6 (21.9,35.3) | 20.2 (12.6,27.9) |
| Shanxi | 37.3 (32.6,42.1) | 34.6 (29.1,40.1) |  | 16.3 (14.5,18.1) | 14.5 (10.7,18.4) |  | 31.2 (26.2,36.1) | 29.7 (24.4,34.9) |
| Anhui | 33.7 (28.2,39.2) | 29.2 (23.2,35.1) |  | 13.7 (11.1,16.3) | 15.1 (11.7,18.5) |  | 28.6 (23.5,33.6) | 22.3 (16.9,27.6) |
| Guangxi | 35.8 (29.9,41.8) | 32.4 (28.5,36.3) |  | 16.0 (13.6,18.4) | 16.7 (12.8,20.7) |  | 30.2 (23.6,36.7) | 24.8 (22.3,27.3) |
| Jiangsu | 31.9 (26.5,37.3) | 27.2 (25.2,29.1) |  | 14.5 (11.4,17.6) | 14.4 (12.8,16.0) |  | 26.2 (21.7,30.6) | 19.9 (18.4,21.3) |
| Guangdong | 36.5 (29.1,43.9) | 31.6 (28.0,35.2) |  | 18.0 (13.2,22.9) | 17.6 (14.2,21.0) |  | 29.2 (23.4,34.9) | 22.7 (19.6,25.8) |
| Tianjin | 36.0 (28.2,43.7) | 35.9 (31.2,40.7) |  | 20.6 (16.9,24.3) | 20.0 (15.8,24.2) |  | 27.7 (20.5,34.8) | 28.0 (23.7,32.3) |
| Shandong | 25.1 (22.3,27.9) | 20.9 (15.5,26.3) |  | 11.6 (9.9,13.3) | 11.0 (8.2,13.8) |  | 19.4 (17.0,21.8) | 15.4 (10.7,20.2) |

Data are presented as %(95%CI).

**Table S4. Prevalence of secondhand smoke exposure (on every day during the past 7 days) by province in Chinese youth from 2013-14 to 2019**

| Province | In any place | |  | At home | |  | In public places | |
| --- | --- | --- | --- | --- | --- | --- | --- | --- |
|  | 2013-14 | 2019 |  | 2013-14 | 2019 |  | 2013-14 | 2019 |
| Yunnan | 41.9 (34.6,49.1) | 29.7 (23.7,35.6) |  | 22.0 (18.5,25.4) | 11.9 (6.3,17.6) |  | 34.2 (27.1,41.3) | 24.3 (19.9,28.8) |
| Neimenggu | 40.3 (31.8,48.7) | 26.2 (24.3,28.1) |  | 21.3 (15.9,26.7) | 15.1 (14.2,16.0) |  | 31.5 (24.2,38.7) | 18.8 (16.5,21.1) |
| Gansu | 37.6 (34.4,40.7) | 31.5 (25.3,37.6) |  | 17.2 (14.6,19.8) | 13.4 (9.9,17.0) |  | 30.9 (28.3,33.5) | 26.0 (20.3,31.8) |
| Shaanxi | 35.5 (29.7,41.4) | 25.5 (21.8,29.1) |  | 13.5 (10.7,16.2) | 10.7 (8.9,12.4) |  | 31.0 (25.5,36.4) | 21.0 (17.6,24.4) |
| Hunan | 34.3 (32.0,36.6) | 34.2 (29.3,39.2) |  | 14.6 (13.0,16.1) | 14.8 (11.2,18.5) |  | 28.8 (26.9,30.7) | 28.4 (23.5,33.3) |
| Hubei | 34.9 (29.6,40.2) | 22.2 (18.7,25.7) |  | 14.4 (12.4,16.4) | 10.7 (8.1,13.3) |  | 29.6 (24.4,34.7) | 17.7 (15.0,20.5) |
| Hainan | 40.9 (35.7,46.0) | 31.0 (27.7,34.3) |  | 18.9 (15.4,22.4) | 16.6 (14.6,18.7) |  | 34.4 (30.2,38.7) | 23.9 (21.0,26.9) |
| Ningxia | 33.3 (27.9,38.7) | 25.9 (22.5,29.4) |  | 13.2 (10.9,15.5) | 9.9 (7.9,11.8) |  | 27.3 (22.1,32.4) | 21.1 (18.0,24.1) |
| Guizhou | 33.7 (28.9,38.5) | 20.7 (17.8,23.5) |  | 15.2 (12.2,18.2) | 11.2 (9.1,13.2) |  | 27.9 (23.2,32.6) | 15.0 (12.0,17.9) |
| Beijing | 35.5 (32.0,39.0) | 28.8 (26.9,30.7) |  | 17.4 (15.3,19.5) | 15.4 (13.2,17.6) |  | 28.5 (25.9,31.2) | 21.0 (19.0,22.9) |
| Jilin | 36.2 (32.0,40.4) | 27.5 (24.7,30.4) |  | 20.5 (17.2,23.7) | 15.1 (13.8,16.3) |  | 26.0 (22.2,29.7) | 20.5 (17.7,23.3) |
| Qinghai | 34.2 (24.6,43.8) | 28.1 (23.2,33.0) |  | 14.5 (11.3,17.7) | 12.3 (9.9,14.7) |  | 28.2 (19.0,37.4) | 22.2 (17.7,26.8) |
| Heilongjiang | 35.5 (33.0,38.0) | 28.3 (24.8,31.8) |  | 18.4 (16.2,20.7) | 15.5 (13.5,17.6) |  | 27.9 (26.0,29.7) | 21.4 (18.5,24.3) |
| Chongqing | 33.5 (28.1,38.9) | 24.3 (22.5,26.2) |  | 13.9 (10.7,17.1) | 11.3 (10.3,12.3) |  | 28.5 (23.6,33.5) | 19.0 (17.4,20.5) |
| Liaoning | 35.2 (30.9,39.4) | 29.3 (24.9,33.7) |  | 18.9 (17.4,20.3) | 15.8 (12.6,19.0) |  | 26.9 (22.3,31.6) | 21.7 (18.4,25.0) |
| Sichuan | 33.7 (28.5,38.9) | 23.6 (21.8,25.4) |  | 15.9 (12.3,19.4) | 11.0 (8.8,13.2) |  | 27.8 (23.4,32.2) | 18.4 (17.1,19.7) |
| Jiangxi | 32.7 (30.1,35.3) | 26.3 (19.9,32.6) |  | 12.7 (10.6,14.9) | 11.2 (9.3,13.1) |  | 27.9 (25.0,30.8) | 21.0 (14.7,27.2) |
| Shanghai | 30.7 (26.9,34.6) | 27.1 (22.3,32.0) |  | 16.2 (14.0,18.4) | 15.9 (13.7,18.1) |  | 21.6 (18.3,24.9) | 17.1 (12.7,21.4) |
| Zhejiang | 29.1 (25.6,32.5) | 24.7 (20.0,29.5) |  | 13.4 (11.3,15.5) | 13.6 (11.4,15.7) |  | 23.4 (20.1,26.7) | 18.1 (13.8,22.3) |
| Fujian | 31.4 (29.5,33.4) | 24.8 (20.6,28.9) |  | 15.9 (13.5,18.2) | 13.1 (11.1,15.2) |  | 24.8 (23.4,26.3) | 18.7 (14.7,22.7) |
| Henan | 27.2 (24.3,30.0) | 20.8 (19.1,22.6) |  | 11.2 (9.2,13.3) | 9.6 (8.8,10.5) |  | 22.2 (20.0,24.4) | 16.1 (14.6,17.6) |
| Hebei | 29.4 (25.1,33.7) | 25.2 (20.3,30.1) |  | 12.8 (10.7,14.9) | 13.8 (10.7,16.9) |  | 23.4 (19.1,27.7) | 18.1 (14.2,21.9) |
| Xinjiang | 27.8 (17.4,38.2) | 18.5 (13.7,23.3) |  | 12.0 (6.0,18.0) | 8.3 (6.0,10.6) |  | 21.8 (14.4,29.3) | 14.0 (9.9,18.1) |
| Xizang | 22.3 (16.7,27.9) | 16.3 (9.7,22.9) |  | 5.5 (4.1,6.9) | 5.0 (2.5,7.4) |  | 20.1 (14.1,26.1) | 13.9 (8.0,19.9) |
| Shanxi | 30.9 (26.5,35.3) | 28.9 (23.6,34.3) |  | 13.2 (11.4,15.0) | 11.8 (8.5,15.2) |  | 25.0 (20.8,29.2) | 24.0 (18.9,29.1) |
| Anhui | 27.3 (23.0,31.6) | 24.1 (18.7,29.5) |  | 11.4 (9.0,13.8) | 12.6 (9.6,15.5) |  | 22.2 (18.4,26.0) | 17.7 (13.2,22.3) |
| Guangxi | 30.3 (24.3,36.4) | 26.9 (23.7,30.0) |  | 13.8 (11.5,16.2) | 14.5 (11.3,17.7) |  | 25.0 (18.9,31.2) | 19.4 (17.5,21.3) |
| Jiangsu | 24.6 (20.1,29.1) | 21.6 (19.8,23.5) |  | 11.6 (9.0,14.2) | 11.7 (10.2,13.1) |  | 19.3 (15.7,22.9) | 15.1 (13.8,16.4) |
| Guangdong | 30.0 (23.7,36.3) | 25.9 (22.9,28.9) |  | 15.0 (11.0,19.1) | 15.3 (12.2,18.5) |  | 23.3 (18.6,28.1) | 17.7 (15.2,20.2) |
| Tianjin | 31.2 (24.9,37.5) | 30.6 (26.4,34.8) |  | 18.2 (15.3,21.0) | 17.6 (13.7,21.5) |  | 22.7 (16.4,29.0) | 22.4 (19.2,25.7) |
| Shandong | 19.7 (17.2,22.3) | 16.8 (12.0,21.7) |  | 9.2 (7.6,10.7) | 9.4 (7.0,11.7) |  | 14.9 (12.8,17.0) | 11.6 (7.4,15.7) |

Data are presented as %(95%CI).

**Table S5. Trends in prevalence of secondhand smoke exposure (on ≥3 days, ≥5 days and daily during the past 7 days) by age, sex, residence, region, GDP per capita category, and status of current cigarette smoking in Chinese adolescents from 2013-14 to 2019**

| **Groups** | ≥3 days | | Absolute change in prevalence | ≥5 days | | Absolute change in prevalence | 7 days | | Absolute change in prevalence |
| --- | --- | --- | --- | --- | --- | --- | --- | --- | --- |
|  | 2013-14 | 2019 |  | 2013-14 | 2019 |  | 2013-14 | 2019 |  |
| **In any place** |  |  |  |  |  |  |  |  |  |
| **All** | 51.1 (49.8,52.5) | 41.7 (40.5,42.9) | -9.5 (-11.3,-7.7) | 38.0 (36.8,39.2) | 30.2 (29.2,31.2) | -7.9 (-9.5,-6.3) | 31.0 (29.9,32.1) | 24.7 (23.8,25.7) | -6.3 (-7.8,-4.9) |
| **Grade** |  |  |  |  |  |  |  |  |  |
| 7th | 43.9 (42.5,45.2) | 35.3 (33.9,36.8) | -8.6 (-10.6,-6.6) | 32.1 (30.9,33.3) | 25.0 (23.9,26.1) | -7.1 (-8.8,-5.4) | 26.1 (25.0,27.1) | 20.4 (19.4,21.4) | -5.7 (-7.2,-4.2) |
| 8th | 52.5 (50.8,54.1) | 43.6 (42.1,45.1) | -8.9 (-11.2,-6.6) | 38.9 (37.4,40.3) | 31.6 (30.3,32.9) | -7.3 (-9.3,-5.3) | 31.6 (30.3,32.9) | 25.8 (24.6,26.9) | -5.9 (-7.6,-4.2) |
| 9th | 56.9 (55.5,58.4) | 46.7 (45.2,48.1) | -10.3 (-12.4,-8.2) | 43.1 (41.7,44.4) | 34.4 (33.0,35.8) | -8.7 (-10.7,-6.7) | 35.3 (34.0,36.6) | 28.4 (27.0,29.7) | -6.9 (-8.8,-5.1) |
| **Sex** |  |  |  |  |  |  |  |  |  |
| Boys | 56.3 (55.0,57.6) | 46.0 (44.6,47.3) | -10.4 (-12.3,-8.4) | 43.0 (41.7,44.3) | 34.0 (32.9,35.2) | -9.0 (-10.8,-7.3) | 35.4 (34.2,36.6) | 28.0 (27.0,29.1) | -7.4 (-9.0,-5.8) |
| Girls | 45.3 (43.9,46.8) | 36.8 (35.6,38.0) | -8.6 (-10.5,-6.7) | 32.5 (31.2,33.7) | 25.8 (24.7,26.8) | -6.7 (-8.3,-5.1) | 26.1 (25.0,27.1) | 20.9 (20.0,21.9) | -5.2 (-6.6,-3.7) |
| **Residence** |  |  |  |  |  |  |  |  |  |
| Urban | 53.1 (51.9,54.4) | 41.7 (40.2,43.3) | -11.4 (-13.6,-9.3) | 40.0 (38.8,41.2) | 30.0 (28.7,31.4) | -10.0 (-11.9,-8.1) | 32.9 (31.8,34.0) | 24.6 (23.4,25.7) | -8.4 (-10.1,-6.7) |
| Rural | 50.4 (48.6,52.1) | 41.7 (40.0,43.3) | -8.7 (-11.2,-6.3) | 37.3 (35.7,38.9) | 30.3 (28.9,31.7) | -7.0 (-9.2,-4.8) | 30.3 (28.9,31.7) | 24.8 (23.5,26.2) | -5.5 (-7.5,-3.5) |
| **Region** |  |  |  |  |  |  |  |  |  |
| North | 52.0 (49.2,54.8) | 43.5 (39.6,47.3) | -8.5 (-13.3,-3.7) | 38.5 (35.7,41.3) | 32.0 (28.5,35.5) | -6.6 (-11.0,-2.0) | 32.0 (29.3,34.7) | 26.6 (23.6,29.7) | -5.4 (-9.5,-1.3) |
| East | 45.1 (43.1,47.1) | 38.4 (35.9,41.0) | -6.7 (-10.0,-3.5) | 32.5 (30.8,34.2) | 27.3 (25.0,29.6) | -5.3 (-8.1,-2.4) | 26.1 (24.6,27.6) | 22.4 (20.3,24.4) | -3.8 (-6.3,-1.2) |
| Central | 52.5 (49.8,55.2) | 42.3 (39.8,44.8) | -10.2 (-13.9,-6.5) | 38.4 (36.1,40.7) | 30.3 (28.1,32.6) | -8.1 (-11.3,-4.8) | 30.5 (28.5,32.5) | 24.9 (22.4,27.3) | -5.7 (-8.8,-2.5) |
| South | 48.7 (42.9,54.4) | 43.4 (40.4,46.5) | -5.3 (-12.0,-1.3) | 37.2 (32.1,42.4) | 32.1 (29.6,34.7) | -5.2 (-11.0,0.7) | 30.9 (26.4,35.4) | 26.5 (24.4,28.6) | -4.4 (-9.4,0.7) |
| Southwest | 56.4 (53.4,59.4) | 42.3 (39.5,45.1) | -14.2 (-18.3,-10.1) | 43.0 (40.0,46.1) | 30.5 (28.3,32.7) | -12.6 (-16.4,-8.7) | 35.5 (32.6,38.4) | 24.4 (22.6,26.1) | -11.1 (-14.6,-7.7) |
| Northwest | 57.0 (53.2,60.7) | 44.3 (40.7,48.0) | -12.6 (-17.9,-7.4) | 42.3 (39.1,45.5) | 31.6 (28.8,34.5) | -10.7 (-15.1,-6.3) | 34.1 (31.0,37.1) | 25.3 (22.9,27.7) | -8.8 (-12.7,-4.8) |
| Northeast | 55.0 (53.0,57.0) | 43.6 (40.5,46.7) | -11.4 (-15.2,-7.7) | 42.3 (40.2,44.4) | 33.7 (31.1,36.3) | -8.6 (-12.0,-5.3) | 35.5 (33.4,37.7) | 28.5 (26.3,30.7) | -7.1 (-10.2,-3.9) |
| **GDP per capita category** |  |  |  |  |  |  |  |  |  |
| Low | 54.5 (52.3,56.6) | 43.9 (41.4,46.4) | -10.6 (-13.9,-7.3) | 41.1 (39.1,43.2) | 32.4 (30.3,34.5) | -8.8 (-11.7,-5.9) | 33.9 (31.9,35.9) | 26.6 (24.8,28.3) | -7.4 (-10.0,-4.7) |
| Middle | 52.6 (50.8,54.4) | 42.7 (41.0,44.4) | -10.0 (-12.5,-7.5) | 39.0 (37.3,40.6) | 30.8 (29.2,32.3) | -8.2 (-10.5,-6.0) | 31.7 (30.3,33.2) | 25.1 (23.6,26.7) | -6.6 (-8.8,-4.5) |
| High | 46.7 (44.1,49.4) | 38.8 (36.7,41.0) | -7.9 (-11.3,-4.5) | 34.5 (32.2,36.9) | 27.9 (26.1,29.7) | -6.7 (-9.7,-3.7) | 27.9 (25.9,29.9) | 22.8 (21.2,24.4) | -5.1 (-7.7,-2.5) |
| **Cigarette smoking** |  |  |  |  |  |  |  |  |  |
| No | 49.0 (47.7,50.4) | 40.2 (39.0,41.4) | -8.9 (-10.7,-7.1) | 36.1 (34.9,37.3) | 28.8 (27.8,29.8) | -7.3 (-8.9,-5.7) | 29.3 (28.2,30.3) | 23.5 (22.6,24.4) | -5.8 (-7.2,-4.4) |
| Yes | 79.8 (78.4,81.1) | 76.3 (74.1,78.5) | -3.5 (-6.15,-0.9) | 65.2 (63.5,66.9) | 62.4 (59.8,65.0) | -2.8 (-5.9,0.3) | 55.0 (53.1,56.8) | 53.0 (50.2,55.7) | -2.1 (-5.4,1.3) |
|  |  |  |  |  |  |  |  |  |  |
| **At home** |  |  |  |  |  |  |  |  |  |
| **All** | 24.4 (23.4,25.3) | 19.8 (19.0,20.5) | -4.6 (-5.8,-3.4) | 17.4 (16.6,18.1) | 14.6 (14.0,15.3) | -2.7 (-3.7,-1.8) | 14.2 (13.6,14.8) | 12.4 (11.8,13.0) | -1.8 (-2.7,-1.0) |
| **Grade** |  |  |  |  |  |  |  |  |  |
| 7th | 21.2 (20.2,22.1) | 17.5 (16.7,18.4) | -3.6 (-4.9,-2.4) | 14.9 (14.1,15.6) | 12.9 (12.2,13.6) | -1.9 (-3.0,-0.9) | 12.1 (11.5,12.7) | 11.0 (10.3,11.6) | -1.1 (-2.1,-0.2) |
| 8th | 25.7 (24.5,26.8) | 20.8 (19.8,21.8) | -4.9 (-6.4,-3.3) | 18.2 (17.3,19.2) | 15.4 (14.6,16.2) | -2.9 (-4.1,-1.6) | 15.0 (14.2,15.7) | 12.9 (12.1,13.7) | -2.1 (-3.2,-1.0) |
| 9th | 26.2 (25.1,27.3) | 21.1 (20.1,22.0) | -5.1 (-6.5,-3.7) | 19.0 (18.1,19.8) | 15.7 (14.9,16.6) | -3.2 (-4.4,-2.0) | 15.5 (14.8,16.3) | 13.4 (12.6,14.2) | -2.2 (-3.3,-1.1) |
| **Sex** |  |  |  |  |  |  |  |  |  |
| Boys | 26.6 (25.6,27.6) | 21.6 (20.8,22.5) | -5.0 (-6.3,-3.7) | 19.0 (18.2,19.8) | 16.0 (15.3,16.7) | -3.0 (-4.0,-1.9) | 15.6 (14.9,16.3) | 13.6 (13.0,14.2) | -2.0 (-2.9,-1.0) |
| Girls | 21.8 (20.9,22.8) | 17.6 (16.8,18.4) | -4.2 (-5.5,-3.0) | 15.5 (14.8,16.2) | 13.0 (12.4,13.7) | -2.5 (-3.5,-1.5) | 12.7 (12.1,13.3) | 11.0 (10.4,11.6) | -1.7 (-2.6,-0.9) |
| **Residence** |  |  |  |  |  |  |  |  |  |
| Urban | 25.7 (24.7,26.7) | 20.2 (19.1,21.3) | -5.5 (-7.1,-4.0) | 18.9 (18.1,19.7) | 15.1 (14.2,16.0) | -3.8 (-5.0,-2.5) | 15.6 (14.9,16.3) | 12.8 (12.0,13.6) | -2.8 (-3.9,-1.8) |
| Rural | 23.8 (24.7,26.0) | 19.5 (18.5,20.5) | -4.3 (-5.9,-2.7) | 16.8 (15.8,17.7) | 14.4 (13.5,15.2) | -2.4 (-3.7,-1.1) | 13.7 (12.9,14.5) | 12.1 (11.4,12.9) | -1.5 (-2.7,-0.4) |
| **Region** |  |  |  |  |  |  |  |  |  |
| North | 25.6 (23.6,27.6) | 21.7 (19.1,24.2) | -3.9 (-7.2,-0.7) | 18.0 (16.4,19.6) | 16.0 (14.0,18.0) | -2.0 (-4.6,0.6) | 14.8 (13.4,16.2) | 13.8 (11.9,15.7) | -1.0 (-3.3,-1.3) |
| East | 20.8 (19.4,22.3) | 18.6 (17.3,19.9) | -2.3 (-4.2,-0.3) | 14.4 (13.4,15.4) | 13.8 (12.6,14.9) | -0.6 (-2.2,0.9) | 11.7 (10.9,12.6) | 11.7 (10.7,12.6) | -0.0 (-1.4,1.2) |
| Central | 23.1 (21.2,25.0) | 18.9 (17.8,20.0) | -4.2 (-6.4,-1.9) | 16.0 (14.5,17.5) | 13.6 (12.7,14.6) | -2.4 (-4.1,-0.6) | 12.7 (11.5,13.9) | 11.3 (10.0,12.6) | -1.4 (-3.2,0.4) |
| South | 23.3 (19.6,27.1) | 22.5 (19.5,25.5) | -0.8 (-5.7,4.1) | 17.8 (14.6,21.0) | 17.4 (14.9,19.8) | -0.4 (-4.5,-3.7) | 15.0 (12.3,17.7) | 15.1 (13.0,17.3) | 0.2 (-3.4,3.7) |
| Southwest | 28.1 (25.7,30.6) | 18.7 (16.5,20.9) | -9.4 (-12.7,-6.1) | 20.4 (18.5,22.2) | 13.5 (11.7,15.3) | -6.9 (-9.5,-4.2) | 16.8 (15.0,18.5) | 11.2 (9.5,12.9) | -5.5 (-8.0,3.1) |
| Northwest | 26.4 (23.6,29.2) | 18.4 (16.1,20.7) | -8.0 (-11.6,-4.3) | 18.1 (15.9,20.3) | 13.3 (11.7,14.9) | -4.8 (-7.5,2.1) | 14.3 (12.5,16.0) | 10.8 (9.5,12.0) | -3.5 (-5.7,1.3) |
| Northeast | 29.9 (28.3,31.4) | 22.8 (21.0,24.6) | -7.1 (-9.5,-4.7) | 22.5 (21.1,24.0) | 18.0 (16.4,19.6) | -4.5 (-6.7,-2.4) | 19.1 (17.8,20.4) | 15.5 (14.1,17.0) | -3.6 (-5.5,1.6) |
| **GDP per capita category** |  |  |  |  |  |  |  |  |  |
| Low | 27.4 (25.9,28.9) | 20.9 (19.0,22.7) | -6.5 (-8.9,-4.1) | 19.5 (18.5,20.6) | 15.5 (14.1,17.0) | -4.0 (-5.8,-2.2) | 16.0 (15.0,17.0) | 13.1 (11.8,14.5) | -2.9 (-4.6,-1.2) |
| Middle | 24.0 (22.6,25.5) | 19.3 (18.4,20.2) | -4.7 (-6.4,-3.1) | 17.0 (15.9,18.1) | 14.0 (13.2,14.8) | -3.0 (-4.3,-1.6) | 13.8 (12.9,14.8) | 11.7 (10.9,12.5) | -2.1 (-3.4,-0.9) |
| High | 22.3 (20.5,24.0) | 19.4 (18.0,20.8) | -2.9 (-5.1,-0.6) | 16.1 (14.6,17.5) | 14.7 (13.5,15.8) | -1.4 (-3.3,0.4) | 13.2 (12.0,14.4) | 12.5 (11.5,13.5) | -0.7 (-2.3,0.9) |
| **Cigarette smoking** |  |  |  |  |  |  |  |  |  |
| No | 23.0 (22.1,23.9) | 18.8 (18.0,19.5) | -4.2 (-5.4,-3.0) | 16.3 (15.6,17.0) | 13.9 (13.3,14.5) | -2.4 (-3.3,-1.5) | 13.3 (12.7,13.9) | 11.7 (11.1,12.2) | -1.6 (-2.4,-0.8) |
| Yes | 43.8 (42.5,45.1) | 42.8 (40.0,45.7) | -1.0 (-4.1,2.2) | 32.5 (31.3,33.7) | 32.5 (29.8,35.2) | 0.0 (-2.9,3.0) | 26.6 (25.2,27.9) | 28.0 (25.2,30.7) | 1.4 (1.7,4.4) |
|  |  |  |  |  |  |  |  |  |  |
| **In public places** |  |  |  |  |  |  |  |  |  |
| **All** | 45.1 (43.8,46.3) | 35.3 (34.1,36.5) | -9.8 (-11.5,-8.1) | 31.8 (30.7,32.8) | 23.8 (22.9,24.8) | -7.9 (-9.4,-6.5) | 25.1 (24.2,26.0) | 18.7 (17.8,19.5) | -6.4 (-7.8,-5.1) |
| **Grade** |  |  |  |  |  |  |  |  |  |
| 7th | 37.6 (36.4,38.8) | 28.4 (27.1,29.7) | -9.2 (-11.1,-7.4) | 26.0 (24.9,27.0) | 18.5 (17.5,19.5) | -7.5 (-9.0,-6.0) | 20.5 (19.5,21.4) | 14.3 (13.5,15.2) | -6.1 (-7.4,-4.9) |
| 8th | 46.1 (44.5,47.7) | 37.1 (35.7,38.5) | -9.0 (-11.2,-6.9) | 32.2 (30.9,33.5) | 25.0 (23.8,26.2) | -7.2 (-9.0,-5.4) | 25.3 (24.2,26.5) | 19.5 (18.5,20.6) | -5.8 (-7.4,-4.3) |
| 9th | 51.3 (49.9,52.7) | 41.0 (39.5,42.4) | -10.4 (-12.5,-8.4) | 37.0 (35.7,38.3) | 28.4 (27.1,29.8) | -8.5 (-10.5,-6.6) | 29.4 (28.2,30.6) | 22.5 (21.3,23.7) | -6.9 (-8.6,-5.2) |
| **Sex** |  |  |  |  |  |  |  |  |  |
| Boys | 50.4 (49.2,51.6) | 39.7 (38.3,41.0) | -10.8 (-12.6,-8.9) | 36.8 (35.6,37.9) | 27.6 (26.5,28.7) | -9.2 (-10.8,-7.6) | 29.4 (28.4,30.5) | 21.8 (20.8,22.9) | -7.6 (-9.1,-6.2) |
| Girls | 39.1 (37.7,40.4) | 30.3 (29.2,31.4) | -8.8 (-10.6,-7.0) | 26.1 (25.0,27.2) | 19.5 (18.6,20.4) | -6.6 (-8.1,-5.2) | 20.2 (19.3,21.1) | 15.0 (14.3,15.8) | -5.2 (-6.4,-3.9) |
| **Residence** |  |  |  |  |  |  |  |  |  |
| Urban | 46.5 (45.3,47.8) | 35.0 (33.5,36.4) | -11.6 (-13.6,-9.5) | 33.1 (31.9,34.2) | 23.2 (22.1,24.3) | -9.8 (-11.5,-8.1) | 26.4 (25.4,27.4) | 18.1 (17.2,19.0) | -8.3 (-9.8,-6.9) |
| Rural | 44.5 (45.3,47.8) | 35.5 (33.9,37.1) | -9.0 (-11.4,-6.7) | 31.3 (29.8,32.7) | 24.2 (22.8,25.5) | -7.1 (-9.1,-5.1) | 24.6 (23.4,25.8) | 19.0 (17.8,20.3) | -5.6 (-7.3,-3.8) |
| **Region** |  |  |  |  |  |  |  |  |  |
| North | 44.9 (42.0,47.9) | 36.4 (32.8,40.1) | -8.5 (-13.2,-3.8) | 31.5 (28.8,34.3) | 25.1 (22.0,28.2) | -6.5 (-10.6,-2.3) | 25.4 (22.9,28.0) | 19.9 (17.2,22.5) | -5.6 (-9.3,-1.9) |
| East | 39.0 (37.2,40.9) | 31.8 (29.3,34.2) | -7.3 (-10.4,-4.2) | 26.7 (25.1,28.2) | 20.8 (18.8,22.8) | -5.9 (-8.4,-3.3) | 20.8 (19.5,22.1) | 16.3 (14.5,18.1) | -4.5 (-6.8,-2.3) |
| Central | 47.1 (37.2,40.9) | 36.9 (34.2,39.5) | -10.2 (-13.9,-6.6) | 32.9 (30.8,35.0) | 24.9 (22.5,27.3) | -8.0 (-11.3-,4.8) | 25.4 (23.7,27.1) | 19.9 (17.4,22.3) | -5.5 (-8.5,-2.5) |
| South | 42.5 (37.5,47.5) | 35.1 (32.5,37.6) | -7.5 (-13.2,-1.8) | 30.4 (26.2,34.7) | 23.8 (21.7,25.8) | -6.7 (-11.5,-1.9) | 24.6 (21.0,28.2) | 18.6 (17.0,20.3) | -6.0 (-10.1,-2.0) |
| Southwest | 50.8 (48.0,53.6) | 36.9 (34.1,39.6) | -14.0 (-17.9,-10.0) | 36.8 (33.9,39.7) | 25.1 (23.1,27.1) | -11.8 (-15.3,-8.2) | 29.4 (26.7,32.1) | 19.1 (17.6,20.5) | -10.3 (-13.4,-7.3) |
| Northwest | 51.5 (48.0,55.0) | 39.4 (35.8,42.9) | -12.2 (-17.2,-7.1) | 36.3 (33.6,39.1) | 26.4 (23.7,29.2) | -9.9 (-13.9,-6.0) | 28.3 (25.9,30.8) | 20.5 (18.3,22.7) | -7.9 (-11.2,-4.5) |
| Northeast | 46.9 (44.5,49.4) | 36.7 (33.7,39.6) | -10.3 (-14.2,-6.4) | 33.5 (31.3,35.7) | 26.3 (24.1,28.6) | -7.2 (-10.4,-4.0) | 27.0 (24.9,29.2) | 21.3 (19.5,23.1) | -5.8 (-8.6,-2.9) |
| **GDP per capita category** |  |  |  |  |  |  |  |  |  |
| Low | 48.0 (45.8,50.2) | 37.5 (35.1,39.8) | -10.6 (-13.8,-7.4) | 34.4 (32.3,36.4) | 25.9 (24.1,27.8) | -8.5 (-11.2,-5.7) | 27.4 (25.5,29.3) | 20.3 (18.8,21.7) | -7.2 (-9.6,-4.8) |
| Middle | 47.1 (45.5,48.7) | 36.8 (35.1,38.6) | -10.3 (-12.7,-7.9) | 33.1 (31.7,34.6) | 24.9 (23.4,26.5) | -8.2 (-10.4,-6.1) | 26.1 (25.5,29.3) | 19.7 (18.1,21.2) | -6.5 (-8.4,-4.5) |
| High | 40.4 (38.0,42.8) | 31.9 (29.9,33.8) | -8.5 (-11.6,-5.5) | 28.1 (26.1,30.1) | 21.0 (19.4,22.5) | -7.2 (-9.7,-4.7) | 22.1 (20.4,23.7) | 16.3 (15.0,17.7) | -5.8 (-7.9,-3.6) |
| **Current cigarette smoking** |  |  |  |  |  |  |  |  |  |
| No | 43.0 (72.3,75.7) | 33.8 (32.7,34.9) | -9.2 (-10.9,-7.5) | 29.9 (28.8,30.9) | 22.5 (21.6,23.4) | -7.4 (-8.8,-6.0) | 23.5 (22.6,24.4) | 17.6 (16.7,18.4) | -5.9 (-7.2,-4.7) |
| Yes | 74.0 (72.3,75.7) | 70.3 (68.0,72.5) | -3.8 (-6.6,-1.0) | 58.2 (56.2,60.3) | 54.9 (52.2,57.6) | -3.4 (-6.7,0.0) | 47.7 (45.6,49.7) | 45.0 (42.1,47.8) | -2.7 (-6.2,0.7) |

Data are presented as proportion (95%CI).

**Abbreviations:** GDP, Gross domestic product.

**Table S6. Trend in prevalence of secondhand smoke exposure by types of public places, age, sex, residence, region, GDP per capita category, and status of current cigarette smoking in Chinese young adolescents from 2013-14 to 2019**

|  | ≥1 day | | Change in prevalence | ≥3 days | | Change in prevalence | ≥5 days | | Change in prevalence | 7 days | | Change in prevalence |
| --- | --- | --- | --- | --- | --- | --- | --- | --- | --- | --- | --- | --- |
|  | 2013-14 | 2019 |  | 2013-14 | 2019 |  | 2013-14 | 2019 |  | 2013-14 | 2019 |  |
| **Closed public places** |  |  |  |  |  |  |  |  |  |  |  |  |
| **All** | 57.2 (55.9,58.6) | 45.8 (44.7,47.0) | -11.5 (-13.2,-9.8) | 34.2 (33.2,35.3) | 26.1 (25.1,27.1) | -8.1 (-9.6,-6.7) | 23.1 (22.3,24.0) | 17.0 (16.1,17.8) | -6.2 (-7.4,-5.0) | 18.1 (17.4,18.8) | 13.2 (12.5,13.9) | -4.9 (-5.9,-3.8) |
| **Grade** |  |  |  |  |  |  |  |  |  |  |  |  |
| 7th | 48.5 (47.0,50.0) | 37.0 (35.5,38.5) | -11.6 (-13.7,-9.5) | 27.4 (26.5,28.4) | 19.7 (18.7,20.8) | -7.7 (-9.1,-6.3) | 18.3 (17.5,19.1) | 12.3 (11.6,13.1) | -5.9 (-7.1,-4.8) | 14.2 (13.5,14.9) | 9.6 (8.9,10.3) | -4.6 (-5.6,-3.7) |
| 8th | 58.5 (56.6,60.4) | 48.6 (47.1,50.1) | -10.0 (-12.4,-7.8) | 34.9 (33.5,36.3) | 27.7 (26.4,28.9) | -7.2 (-9.1,-5.4) | 23.4 (22.4,24.5) | 18.0 (17.0,19.0) | -5.4 (-6.9,-3.9) | 18.2 (17.3,19.2) | 14.0 (13.2,14.9) | -4.2 (-5.5,-2.9) |
| 9th | 64.6 (63.3,65.9) | 52.6 (51.3,53.9) | -12.0 (-13.9,-10.2) | 40.3 (38.9,41.6) | 31.5 (30.2,32.7) | -8.8 (-10.7,-6.9) | 27.6 (26.4,28.7) | 20.9 (19.7,22.0) | -6.7 (-8.3,-5.0) | 21.8 (20.8,22.7) | 16.4 (15.4,17.4) | -5.4 (-6.8,-4.0) |
| **Sex** |  |  |  |  |  |  |  |  |  |  |  |  |
| Boys | 60.2 (58.9,61.5) | 47.9 (46.6,49.1) | -12.4 (-14.3,-10.7) | 38.7 (37.6,39.8) | 29.5 (28.3,30.6) | -9.2 (-10.9,-7.6) | 26.9 (25.9,27.9) | 19.6 (18.6,20.6) | -7.3 (-8.7,-5.9) | 21.4 (20.5,22.2) | 15.4 (14.5,16.3) | -6.0 (-7.2,-4.7) |
| Girls | 53.9 (52.4,55.3) | 43.5 (42.3,44.7) | -10.5 (-12.4,-8.6) | 29.2 (28.1,30.3) | 22.3 (21.3,23.2) | -7.0 (-8.4,-5.5) | 18.8 (18.0,19.7) | 13.9 (13.2,14.6) | -5.0 (-6.1,-3.8) | 14.4 (13.7,15.1) | 10.7 (10.1,11.4) | -3.7 (-4.6,-2.7) |
| **Residence** |  |  |  |  |  |  |  |  |  |  |  |  |
| Urban | 57.3 (56.0,58.5) | 44.9 (43.4,46.4) | -12.5 (-14.4,-10.5) | 33.9 (32.6,35.1) | 24.5 (23.5,25.6) | -9.3 (-11.1,-7.6) | 22.8 (21.7,23.8) | 15.7 (15.0,16.5) | -7.0 (-8.4,-5.6) | 18.1 (17.1,19.0) | 12.2 (11.6,12.8) | -5.9 (-7.0,-4.7) |
| Rural | 57.2 (55.4,59.0) | 46.3 (44.8,47.9) | -11.0 (-13.3,-8.6) | 34.4 (33.0,35.8) | 27.0 (25.6,28.4) | -7.4 (-9.4,-5.4) | 23.3 (22.2,24.4) | 17.6 (16.5,18.8) | -5.6 (-7.3,-4.0) | 18.1 (17.2,19.0) | 13.8 (12.8,14.9) | -4.3 (-5.7,-2.9) |
| **Region** |  |  |  |  |  |  |  |  |  |  |  |  |
| North | 56.6 (53.7,59.6) | 46.0 (42.4,49.7) | -10.6 (-15.3,-5.8) | 33.8 (31.0,36.7) | 27.2 (24.1,30.2) | -6.7 (-10.9,-2.5) | 22.6 (20.1,25.2) | 18.0 (15.4,20.6) | -4.7 (-8.3,-1.0) | 18.0 (15.7,20.4) | 14.2 (12.2,16.3) | -3.8 (-7.0,0.7) |
| East | 52.4 (50.5,54.3) | 42.4 (40.1,44.8) | -9.9 (-13.0,-6.9) | 28.9 (27.5,30.3) | 23.0 (21.2,24.9) | -5.9 (-8.2,-3.6) | 18.9 (17.8,20.0) | 14.6 (13.0,16.2) | -4.3 (-6.3,-2.3) | 14.7 (13.7,15.6) | 11.4 (10.0,12.8) | -3.3 (-5.0,-1.6) |
| Central | 61.2 (58.7,63.7) | 47.9 (45.6,50.1) | -13.3 (-16.7,-9.9) | 37.0 (34.7,39.3) | 28.1 (25.7,30.5) | -9.0 (-12.3,-5.6) | 24.7 (22.9,26.5) | 18.2 (16.0,20.3) | -6.5 (-9.3,-3.7) | 18.9 (17.5,20.4) | 14.7 (12.4,16.9) | -4.3 (-6.9,-1.6) |
| South | 52.1 (46.6,57.7) | 46.6 (44.1,49.2) | -5.5 (-11.7,0.7) | 31.8 (27.7,35.9) | 26.0 (23.7,28.2) | -5.8 (-10.6,-1.1) | 22.2 (18.9,25.4) | 16.9 (15.3,18.5) | -5.3 (-9.0,-1.6) | 17.8 (15.1,20.5) | 13.1 (12.0,14.2) | -4.8 (-7.7,-1.8) |
| Southwest | 62.9 (60.4,65.5) | 48.0 (45.0,51.1) | -14.9 (-18.9,-10.9) | 40.1 (37.5,42.7) | 27.3 (25.0,29.7) | -12.8 (-16.3,-9.2) | 28.0 (25.6,30.3) | 17.8 (16.0,19.6) | -10.2 (-13.1,-7.2) | 21.9 (19.8,24) | 13.5 (12.2,14.9) | -8.4 (-10.9,-5.8) |
| Northwest | 63.9 (61.0,66.8) | 49.9 (46.3,53.5) | -14.0 (-18.7,-9.3) | 38.8 (36.0,41.6) | 29.2 (26.1,32.2) | -9.6 (-13.9,-5.4) | 25.9 (23.7,28.2) | 18.9 (16.8,21.1) | -7.0 (-10.2,-3.8) | 20.0 (18.1,21.8) | 14.2 (12.6,15.9) | -5.7 (-8.3,-3.2) |
| Northeast | 57.2 (54.8,59.6) | 42.4 (39.2,45.7) | -14.8 (-18.9,-10.7) | 33.5 (31.3,35.7) | 26.2 (23.8,28.6) | -7.3 (-10.6,-4.0) | 22.8 (21.0,24.6) | 18.0 (16.4,19.6) | -4.8 (-7.3,-2.3) | 18.2 (16.6,19.9) | 14.3 (13.0,15.6) | -3.9 (-6.0,-1.8) |
| **GDP per capita category** |  |  |  |  |  |  |  |  |  |  |  |  |
| Low | 59.8 (57.6,62.0) | 48.4 (45.9,51.0) | -11.4 (-14.7,-8.0) | 36.9 (34.9,38.9) | 28.4 (26.4,30.3) | -8.5 (-11.3,-5.7) | 25.4 (23.6,27.1) | 18.9 (17.2,20.5) | -6.5 (-8.9,-4.1) | 20.0 (18.5,21.6) | 14.7 (13.5,15.9) | -5.3 (-7.3,-3.4) |
| Middle | 59.7 (58.1,61.3) | 47.3 (45.6,48.9) | -12.5 (-14.8,-10.1) | 36.1 (34.7,37.5) | 27.6 (26.0,29.1) | -8.5 (-10.6,-6.4) | 24.3 (23.1,25.4) | 17.7 (16.3,19.1) | -6.5 (-8.4,-4.7) | 18.9 (17.9,19.9) | 13.9 (12.5,15.3) | -5.0 (-6.7,-3.3) |
| High | 52.7 (50.1,55.3) | 42.2 (40.4,44.0) | -10.5 (-13.7,-7.4) | 30.0 (28.1,32.0) | 22.7 (21.2,24.2) | -7.3 (-9.8,-4.9) | 20.0 (18.5,21.5) | 14.5 (13.4,15.7) | -5.5 (-7.4,-3.6) | 15.6 (14.4,16.8) | 11.3 (10.4,12.2) | -4.3 (-5.8,-2.8) |
| **Current cigarette smoking** |  |  |  |  |  |  |  |  |  |  |  |  |
| No | 55.4 (54.1,56.7) | 44.3 (43.2,45.4) | -11.1 (-12.8,-9.4) | 32.2 (31.2,33.2) | 24.6 (23.7,25.5) | -7.6 (-9.0,-6.2) | 21.4 (20.6,22.2) | 15.7 (15.0,16.5) | -5.7 (-6.8,-4.5) | 16.6 (16.0,17.3) | 12.3 (11.6,12.9) | -4.4 (-5.3,-3.4) |
| Yes | 84.4 (83.0,85.8) | 82.1 (80.1,84.1) | -2.3 (-4.8,0.2) | 62.9 (61.1,64.7) | 60.8 (58.4,63.2) | -2.1 (-5.2,0.9) | 47.1 (45.2,49.0) | 45.1 (42.3,48.0) | -1.9 (-5.4,1.5) | 38.4 (36.5,40.3) | 36.0 (33.0,38.9) | -2.4 (-6.0,-1.1) |
| **Open public places** |  |  |  |  |  |  |  |  |  |  |  |  |
| **All** | 58.3 (56.9,59.7) | 48.6 (47.4,49.9) | -9.8 (-11.6,-7.9) | 35.3 (34.2,36.4) | 27.9 (26.8,29.0) | -7.3 (-8.9,-5.8) | 23.9 (23.0,24.8) | 18.5 (17.7,19.3) | -5.4 (-6.7,-4.2) | 18.5 (17.8,19.3) | 14.4 (13.7,15.1) | -4.2 (-5.2,-3.1) |
| **Grade** |  |  |  |  |  |  |  |  |  |  |  |  |
| 7th | 50.1 (48.5,51.6) | 40.5 (38.9,42.2) | -9.6 (-11.9,-7.4) | 28.5 (27.5,29.6) | 21.9 (20.7,23.0) | -6.6 (-8.2,-5.1) | 19.3 (18.3,20.2) | 14.2 (13.3,15.0) | -5.1 (-6.3,-3.8) | 14.8 (14.0,15.6) | 11.0 (10.3,11.7) | -3.9 (-4.9,-2.8) |
| 8th | 59.4 (57.5,61.2) | 51.0 (49.4,52.6) | -8.5 (-10.8,-6.1) | 35.9 (34.5,37.3) | 29.2 (27.8,30.6) | -6.7 (-8.7,-4.7) | 23.9 (22.8,24.9) | 19.2 (18.2,20.2) | -4.7 (-6.2,-3.2) | 18.4 (17.5,19.4) | 14.9 (14.0,15.7) | -3.6 (-4.9,-2.3) |
| 9th | 65.4 (64.1,66.6) | 55.0 (53.6,56.5) | -10.4 (-12.3,-8.4) | 41.2 (40.0,42.5) | 33.2 (31.8,34.5) | -8.1 (-10.0,-6.2) | 28.6 (27.5,29.7) | 22.6 (21.4,23.7) | -6.0 (-7.7,-4.4) | 22.3 (21.3,23.3) | 17.6 (16.6,18.6) | -4.7 (-6.1,-3.3) |
| **Sex** |  |  |  |  |  |  |  |  |  |  |  |  |
| Boys | 61.0 (69.7,62.3) | 50.6 (49.2,52.0) | -10.5 (-12.4,-8.6) | 39.4 (38.4,40.5) | 31.5 (30.2,32.8) | -7.9 (-9.6,-6.2) | 27.6 (26.6,28.5) | 21.4 (20.5,22.4) | -6.1 (-7.5,-4.7) | 21.6 (20.8,22.5) | 16.8 (16.0,17.6) | -4.8 (-6.0,-3.6) |
| Girls | 55.3 (53.8,56.8) | 46.4 (45.1,47.7) | -9.0 (-11.0,-7.0) | 30.6 (29.4,31.8) | 23.8 (22.8,24.8) | -6.8 (-8.4,-5.2) | 19.9 (19.0,20.8) | 15.1 (14.3,15.9) | -4.7 (-6.0,-3.5) | 15.1 (14.3,15.8) | 11.6 (10.9,12.3) | -3.5 (-4.5,-2.4) |
| **Residence** |  |  |  |  |  |  |  |  |  |  |  |  |
| Urban | 60.3 (59.0,61.6) | 49.5 (47.6,51.4) | -10.9 (-13.2,-8.6) | 37.7 (36.5,38.8) | 28.2 (26.9,29.6) | -9.4 (-11.3,-7.5) | 26.1 (25.2,27.0) | 18.4 (17.5,19.4) | -7.7 (-9.1,-6.2) | 20.6 (19.8,21.3) | 14.3 (13.5,15.1) | -6.3 (-7.4,-5.1) |
| Rural | 57.5 (55.7,59.3) | 48.2 (46.5,49.9) | -9.4 (-11.9,-7.0) | 34.3 (32.9,35.8) | 27.7 (26.2,29.2) | -6.6 (-8.7,-4.5) | 23.1 (21.9,24.3) | 18.5 (17.4,19.7) | -4.5 (-6.2,-2.9) | 17.8 (16.7,18.8) | 14.4 (13.4,15.4) | -3.3 (-4.8,-1.9) |
| **Region** |  |  |  |  |  |  |  |  |  |  |  |  |
| North | 57.6 (55.0,60.3) | 47.8 (43.8,51.7) | -9.9 (-14.7,-5.1) | 35.5 (33.3,37.8) | 29.1 (25.8,32.3) | -6.5 (-10.4,-2.5) | 23.8 (21.7,25.9) | 19.4 (16.9,22.0) | -4.4 (-7.7,-1.0) | 18.5 (16.7,20.3) | 15.2 (13.2,17.3) | -3.3 (-6.0,-0.5) |
| East | 53.7 (51.8,55.7) | 45.7 (42.9,48.5) | -8.0 (-11.5,-4.5) | 30.3 (28.7,31.9) | 25.3 (23.1,27.6) | -4.9 (-7.7,-2.2) | 20.0 (18.8,21.3) | 16.5 (14.7,18.2) | -3.6 (-5.7,-1.4) | 15.4 (14.4,16.5) | 12.8 (11.3,14.3) | -2.6 (-4.5,-0.7) |
| Central | 60.7 (58.4,63.1) | 49.2 (46.7,51.7) | -11.5 (-15.0,-8.1) | 35.7 (33.6,37.8) | 28.7 (26.0,31.3) | -7.1 (-10.5,-3.7) | 24.2 (22.5,26.0) | 19.0 (16.9,21.0) | -5.3 (-8.0,-2.6) | 18.5 (17.1,20.0) | 15.0 (13.0,16.9) | -3.6 (-6.1,-1.1) |
| South | 53.9 (48.1,59.7) | 50.6 (47.5,53.7) | -3.3 (-10.0,3.5) | 33.4 (28.8,37.9) | 28.3 (25.7,30.9) | -5.1 (-10.4,0.3) | 23.1 (19.5,26.6) | 18.9 (17.2,20.6) | -4.2 (-8.2,-0.1) | 18.4 (15.2,21.5) | 14.9 (13.6,16.2) | -3.5 (-6.9,0.0) |
| Southwest | 63.7 (61.1,66.3) | 50.6 (47.4,53.8) | -13.1 (-17.2,-8.9) | 40.1 (37.3,42.9) | 28.4 (26.1,30.6) | -11.7 (-15.4,8.1) | 27.8 (25.1,30.4) | 18.7 (17.1,20.3) | -9.1 (-12.2,-5.9) | 21.5 (19.2,23.8) | 14.0 (12.8,15.2) | -7.5 (-10.1,-4.9) |
| Northwest | 65.4 (61.8,69.1) | 53.1 (49.4,56.8) | -12.4 (-17.6,-7.1) | 41.1 (38.0,44.1) | 31.2 (28.0,34.3) | -9.9 (-14.4,-5.4) | 27.7 (25.2,30.2) | 20.7 (18.2,23.1) | -7.0 (-10.6,-3.5) | 20.7 (18.6,22.9) | 15.6 (13.8,17.5) | -5.1 (-8.0,-2.3) |
| Northeast | 59.9 (57.3,62.6) | 47.4 (44.2,50.5) | -12.6 (-16.7,-8.4) | 38.1 (35.8,40.4) | 29.9 (27.3,32.5) | -8.2 (-11.8,-4.7) | 26.5 (24.4,28.5) | 21.3 (19.2,23.5) | -5.1 (-8.1,-2.1) | 21.1 (19.2,23.0) | 17.1 (15.3,18.9) | -4.0 (-6.7,-1.4) |
| **GDP per capita category** |  |  |  |  |  |  |  |  |  |  |  |  |
| Low | 61.1 (58.9,63.3) | 50.5 (47.9,53.2) | -10.6 (-14.0,-7.1) | 38.2 (36.1,40.2) | 29.4 (27.4,31.4) | -8.8 (-11.7,-5.9) | 26.1 (24.3,27.9) | 19.8 (18.3,21.3) | -6.2 (-8.6,-3.9) | 20.1 (18.5,21.6) | 15.3 (14.1,16.4) | -4.8 (-6.8,-2.8) |
| Middle | 60.3 (58.6,62.0) | 49.4 (47.6,51.2) | -10.9 (-13.3,-8.4) | 36.5 (35.0,37.9) | 28.9 (27.2,30.5) | -7.6 (-9.8,-5.4) | 24.7 (23.5,26.0) | 19.2 (17.9,20.6) | -5.5 (-7.3,-3.7) | 19.2 (18.1,20.2) | 15.0 (13.8,16.2) | -4.2 (-5.8,-2.5) |
| High | 54.1 (51.4,56.8) | 46.3 (44.0,48.6) | -7.8 (-11.3,-4.2) | 31.6 (29.5,33.7) | 25.7 (23.9,27.6) | -5.9 (-8.7,-3.0) | 21.3 (19.7,22.9) | 16.6 (15.3,18.0) | -4.7 (-6.8,-2.5) | 16.6 (15.2,18.0) | 13.0 (11.9,14.1) | -3.6 (-5.4,-1.8) |
| **Current cigarette smoking** |  |  |  |  |  |  |  |  |  |  |  |  |
| No | 56.7 (55.4,58.0) | 47.3 (46.0,48.6) | -9.4 (-11.2,-7.5) | 33.5 (32.4,34.5) | 26.7 (25.6,27.7) | -6.8 (-8.3,-5.3) | 22.6 (21.7,23.4) | 17.5 (16.7,18.2) | -5.1 (-6.3,-3.9) | 17.4 (16.6,18.1) | 13.5 (12.9,14.2) | -3.9 (-4.9,-2.8) |
| Yes | 82.4 (80.8,84.0) | 80.0 (77.7,82.3) | -2.4 (-5.3,0.4) | 60.2 (58.2,62.2) | 57.2 (54.5,60.0) | -3.0 (-6.4,0.4) | 43.5 (41.4,45.5) | 43.1 (40.5,45.7) | -0.3 (-3.6,3.0) | 34.6 (32.8,36.5) | 34.4 (31.7,37.1) | -0.2 (-3.5,3.1) |
| **Public transportation** |  |  |  |  |  |  |  |  |  |  |  |  |
| **All** | 37.9 (36.8,39.0) | 23.4 (22.3,24.4) | -14.6 (-16.2,-13.1) | 16.3 (15.6,17.0) | 10.0 (9.4,10.6) | -6.3 (-7.3,-5.4) | 10.4 (9.8,10.9) | 6.3 (5.8,6.8) | -4.0 (-4.8,-3.3) | 7.7 (7.3,8.2) | 4.7 (4.3,5.1) | -3.0 (-3.6,-2.4) |
| **Grade** |  |  |  |  |  |  |  |  |  |  |  |  |
| 7th | 31.9 (30.6,33.2) | 18.8 (17.5,20.1) | -13.2 (-15.1,-11.3) | 12.6 (11.9,13.3) | 7.7 (7.0,8.4) | -4.9 (-5.9,-3.9) | 7.7 (7.2,8.3) | 4.8 (4.3,5.4) | -2.9 (-3.7,-2.1) | 5.8 (5.3,6.3) | 3.4 (3.0,3.8) | -2.4 (-3.0,-1.7) |
| 8th | 38.4 (37.0,39.8) | 23.7 (22.2,25.2) | -14.8 (-16.9,-12.8) | 16.9 (16.0,17.8) | 9.8 (9.0,10.6) | -7.1 (-8.3,-5.9) | 10.7 (10.0,11.5) | 6.2 (5.6,6.9) | -4.5 (-5.5,-3.5) | 8.0 (7.5,8.6) | 4.7 (4.2,5.2) | -3.3 (-4.1,-2.5) |
| 9th | 43.1 (41.8,44.5) | 28.0 (26.9,29.1) | -15.2 (-17.0,-13.4) | 19.2 (18.3,20.2) | 12.6 (11.7,13.4) | -6.7 (-7.9,-5.4) | 12.5 (11.7,13.3) | 8.0 (7.4,8.7) | -4.5 (-5.5,-3.4) | 9.3 (8.7,10.0) | 6.1 (5.6,6.6) | -3.2 (-4.0,-2.4) |
| **Sex** |  |  |  |  |  |  |  |  |  |  |  |  |
| Boys | 40.9 (39.7,42.0) | 25.9 (24.7,27.0) | -15.1 (-16.8,-13.4) | 19.1 (18.3,20.0) | 12.1 (11.3,12.8) | -7.0 (-8.2,-5.9) | 12.5 (11.8,13.1) | 8.0 (7.3,8.6) | -4.5 (-5.4,-3.5) | 9.4 (8.8,9.9) | 6.0 (5.5,6.5) | -3.3 (-4.1,-2.6) |
| Girls | 34.5 (33.3,35.7) | 20.5 (19.3,21.6) | -14.1 (-15.8,-12.4) | 13.0 (12.3,13.8) | 7.5  (6.9,8.0) | -5.6 (-6.5,-4.6) | 8.0 (7.4,8.5) | 4.4 (4.0,4.8) | -3.6 (-4.3,-2.9) | 5.9 (5.4,6.3) | 3.2 (2.8,3.5) | -2.7 (-3.3,-2.1) |
| **Residence** |  |  |  |  |  |  |  |  |  |  |  |  |
| Urban | 36.1 (34.8,37.4) | 20.7 (19.5,21.9) | -15.4 (-17.3,-13.5) | 16.4 (15.5,17.2) | 9.2 (8.5,9.9) | -7.2 (-8.3,-6.0) | 10.3 (9.6,11.0) | 5.7 (5.3,6.1) | -4.6 (-5.4,-3.8) | 7.7 (7.2,8.2) | 4.3 (3.9,4.6) | -3.5 (-4.1,-2.8) |
| Rural | 38.8 (37.3,40.2) | 25.1 (23.7,26.5) | -13.7 (-15.9,-11.6) | 16.3 (15.3,17.2) | 10.5 (9.6,11.3) | -5.8 (-7.1,-4.5) | 10.4 (9.7,11.1) | 6.7 (6.0,7.5) | -3.7 (-4.7,-2.6) | 7.8 (7.1,8.4) | 5.0 (4.4,5.6) | -2.7 (-3.6,-1.9) |
| **Region** |  |  |  |  |  |  |  |  |  |  |  |  |
| North | 38.5 (35.8,41.2) | 23.6 (21.1,26.1) | -14.9 (-18.6,-11.3) | 17.0 (14.7,19.3) | 11.2 (9.1,13.2) | -5.8 (-9.0,-2.7) | 11.3 (9.5,13.1) | 7.1 (5.6,8.5) | -4.2 (-6.5,-1.9) | 8.7 (7.2,10.2) | 5.1 (3.9,6.4) | -3.6 (-5.5,-1.6) |
| East | 31.5 (29.7,33.3) | 19.2 (17.6,20.8) | -12.3 (-14.7,-9.9) | 13.0 (11.9,14.0) | 8.0 (7.2,8.9) | -5.0 (-6.3,-3.6) | 8.1 (7.3,8.9) | 5.2 (4.6,5.8) | -2.9 (-3.9,-1.9) | 6.0 (5.4,6.6) | 3.8 (3.3,4.3) | -2.2 (-3.0,-1.4) |
| Central | 44.0 (41.6,46.4) | 26.7 (23.8,29.7) | -17.3 (-21.1,-13.4) | 18.7 (16.8,20.6) | 12.0 (10.0,14.0) | -6.8 (-9.6,-4.0) | 11.8 (10.4,13.1) | 7.8 (5.8,9.8) | -3.9 (-6.4,-1.5) | 8.7 (7.5,9.9) | 5.8 (4.4,7.2) | -2.9 (-4.8,-1.1) |
| South | 28.9 (26.2,31.7) | 18.6 (15.9,21.2) | -10.4 (-14.3,-6.5) | 12.7 (11.2,14.1) | 8.3 (6.8,9.8) | -4.4 (-6.6,-2.3) | 8.1 (6.9,9.2) | 5.5 (4.6,6.4) | -2.6 (-4.1,-1.1) | 6.2 (5.3,7.1) | 4.2 (3.4,5.0) | -2.0 (-3.2,-0.8) |
| Southwest | 44.3 (41.2,47.3) | 27.4 (25.0,29.8) | -16.8 (-20.7,-12.9) | 18.5 (16.3,20.7) | 9.8 (8.9,10.7) | -8.7 (-11.1,-6.3) | 12.1 (10.3,13.9) | 6.0 (5.6,6.5) | -6.1 (-8.0,-4.2) | 9.0 (7.5,10.5) | 4.6 (4.2,5.1) | -4.4 (-5.9,-2.8) |
| Northwest | 43.5 (40.0,47.1) | 25.9 (23.4,28.4) | -17.7 (-22.1,-13.3) | 19.4 (17.2,21.5) | 11.2 (9.3,13.0) | -8.2 (-11.1,-5.4) | 11.7 (10.4,13.1) | 6.3 (5.0,7.6) | -5.4 (-7.3,-3.6) | 8.5 (7.4,9.6) | 4.7 (3.7,5.7) | -3.8 (-5.3,-2.2) |
| Northeast | 39.6 (36.7,42.4) | 24.7 (22.0,27.3) | -14.9 (-18.9,-10.9) | 18.1 (16.3,20.0) | 12.3 (10.6,14.0) | -5.8 (-8.4,-3.3) | 11.5 (10,12.9) | 7.8 (6.6,9.0) | -3.7 (-5.5,-1.8) | 8.7 (7.5,10.0) | 5.8 (4.9,6.7) | -3.0 (-4.5,-1.4) |
| **GDP per capita category** |  |  |  |  |  |  |  |  |  |  |  |  |
| Low | 42.1 (39.9,44.3) | 25.7 (24.2,27.2) | -16.4 (-19.1,-13.7) | 18.1 (16.7,19.5) | 10.8 (9.8,11.8) | -7.3 (-9.0,-5.5) | 11.6 (10.6,12.6) | 6.8 (6.1,7.5) | -4.8 (-6.0,-3.6) | 8.5 (7.7,9.4) | 5.1 (4.5,5.7) | -3.5 (-4.5,-2.4) |
| Middle | 41.5 (39.8,43.3) | 25.7 (23.7,27.6) | -15.9 (-18.5,-13.3) | 18.1 (16.8,19.4) | 10.8 (9.6,11.9) | -7.3 (-9.0,-5.5) | 11.4 (10.4,12.4) | 6.8 (5.7,7.8) | -4.6 (-6.1,-3.2) | 8.6 (7.8,9.5) | 5.1 (4.3,5.9) | -3.5 (-4.7,-2.4) |
| High | 30.5 (28.8,32.1) | 18.9 (17.4,20.3) | -11.6 (-13.8,-9.4) | 12.8 (11.9,13.7) | 8.3  (7.6,9.0) | -4.5 (-5.6,-3.3) | 8.1 (7.4,8.8) | 5.4 (4.8,5.9) | -2.7 (-3.6,-1.8) | 6.1 (5.5,6.6) | 4.0 (3.6,4.4) | -2.1 (-2.8,-1.4) |
| **Current cigarette smoking** |  |  |  |  |  |  |  |  |  |  |  |  |
| No | 36.3 (35.2,37.4) | 22.2 (21.2,23.2) | -14.1 (-15.7,-12.6) | 14.9 (14.2,15.6) | 9.0  (8.4,9.6) | -5.9 (-6.8,-5.0) | 9.3 (8.8,9.9) | 5.6 (5.2,6.1) | -3.7 (-4.4,-3.0) | 7.0 (6.6,7.4) | 4.2 (3.8,4.5) | -2.9 (-3.4,-2.3) |
| Yes | 56.1 (53.5,58.7) | 45.4 (42.6,48.2) | -10.7 (-6.9,-14.5) | 30.9 (28.7,33.0) | 27.0 (24.3,29.8) | -3.8 (-7.3,-0.4) | 20.9 (18.9,22.9) | 19.2 (16.5,21.9) | -1.7 (-5.0,1.7) | 15.5 (14.0,17.0) | 14.7 (12.6,16.8) | -0.8 (-3.3,1.8) |

Data are presented as %(95%CI).

**Table S7. Trend in prevalence of secondhand smoke exposure (on ≥1 day during the past 30 days) in schools by age, sex, residence, region, GDP per capita category, and status of current cigarette smoking in Chinese young adolescents from 2013-14 to 2019**

|  | 2013-14 | 2019 | Change in prevalence |
| --- | --- | --- | --- |
| **All** | 54.5(52.8, 56.2) | 45.2(43.7, 46.8) | -9.3(-11.6,-7.0) |
| **Grade** |  |  |  |
| 7th | 47.4(45.7, 49.2) | 37.5(35.7, 39.3) | -10.0(-12.6,-7.5) |
| 8th | 55.3(53.2, 57.4) | 46.7(44.9, 48.5) | -8.7(-11.4,-5.9) |
| 9th | 60.6(58.7, 62.6) | 52.2(50.5, 53.9) | -8.4(-10.9,-5.8) |
| **Sex** |  |  |  |
| Boys | 60.4(58.8, 62.1) | 50.2(48.6, 51.9) | -10.3(-12.6,-7.9) |
| Girls | 47.8(45.9, 49.6) | 39.5(37.9, 41.0) | -8.3(-10.7,-5.8) |
| **Residence** |  |  |  |
| Urban | 50.7(48.2, 53.2) | 38.1(36.3, 39.9) | -12.7(-15.8,-9.5) |
| Rural | 55.9(53.7, 58.1) | 49.3(47.2, 51.4) | -6.6(-9.7,-3.6) |
| **Region** |  |  |  |
| North | 49.5(45.8,53.2) | 43.1(38.7, 47.6) | -6.4(-12.2,-0.6) |
| East | 48.9(46.4,51.3) | 39.8(37.0, 42.5) | -9.1(-12.8,-5.4) |
| Central | 60.8(57.0,64.7) | 48.2(45.2, 51.2) | -12.6(-17.6,-7.7) |
| South | 53.0(46.5,59.5) | 47.2(42.4, 52.0) | -5.8(-14.0,-2.5) |
| Southwest | 62.1(58.0,66.1) | 53.5(49.2, 57.8) | -8.6(-14.5,-2.6) |
| Northwest | 58.9(54.3,63.6) | 45.9(41.5, 50.2) | -13.1(-19.5,-6.6) |
| Northeast | 46.2(42.5,49.9) | 38.0(34.3, 41.8) | -8.2(-13.5,-2.8) |
| **GDP per capita category** | |  |  |
| Low | 57.1(54.2,60.0) | 49.7(46.4, 52.9) | -7.4(-11.8,-3.1) |
| Middle | 58.1(55.7,60.5) | 48.3(46.1, 50.5) | -9.8(-13.1,-6.6) |
| High | 48.3(45.2,51.5) | 38.3(35.8, 40.8) | -10.0(-14.0,-6.0) |
| **Current cigarette smoking** | |  |  |
| No | 52.4(50.7,54.0) | 43.6(42.2, 45.1) | -8.7(-11.0,-6.5) |
| Yes | 83.4(81.7,85.1) | 81.0(79.0, 83.1) | -2.3(-5.0,0.4) |

Data are presented as %(95%CI).

**
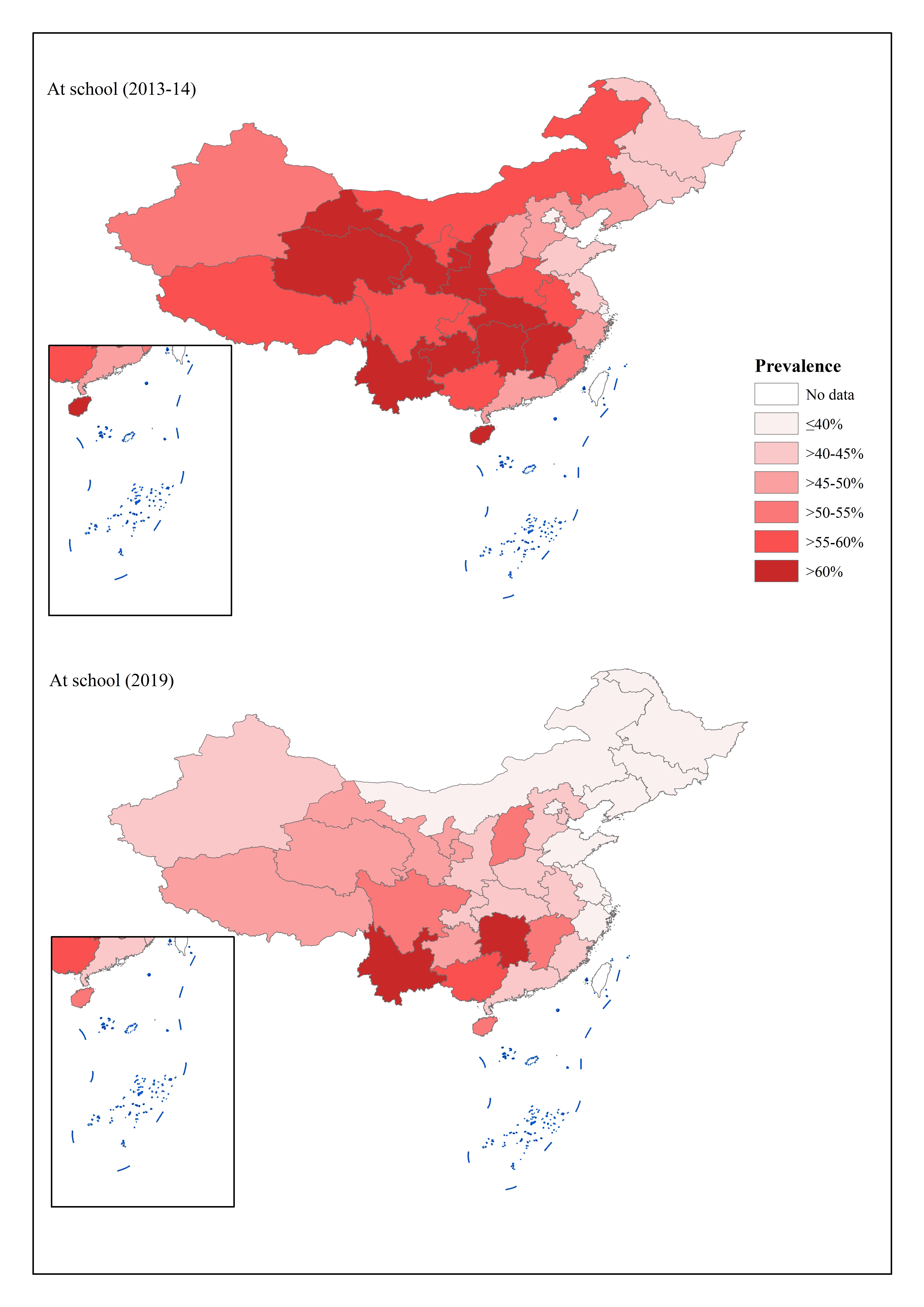
**

**Figure S1:** Trends of geographical distributions of secondhand smoke exposure (on ≥1 day during the past 30 days) at school in Chinese young adolescents from 2013-14 to 2019

**Table S8. Trend in attitude on hazards of secondhand smoke exposure by age, sex, residence, region, GDP per capita category, and status of current cigarette smoking in Chinese young adolescents from 2013-2014 to 2019**

|  | 2013-14 | | | |  | 2019 | | | |
| --- | --- | --- | --- | --- | --- | --- | --- | --- | --- |
|  | Definitely not | Probably not | Might be | Yes |  | Definitely not | Probably not | Might be | Yes |
| **All** | 2.6(2.4, 2.8) | 2.4(2.2, 2.5) | 21.1(20.6, 21.7) | 73.9(73.3, 74.5) |  | 2.2(2.0, 2.4) | 1.8(1.6, 2.0) | 22.3(21.5, 23.1) | 73.7(72.7, 74.8) |
| **Grade** |  |  |  |  |  |  |  |  |  |
| 7th | 3.3(2.8, 3.7) | 2.6(2.3, 2.9) | 23.3(22.5, 24.1) | 70.9(70.0, 71.8) |  | 2.4(2.2, 2.7) | 1.9(1.6, 2.2) | 24.0(22.9, 25.1) | 71.7(70.3, 73.0) |
| 8th | 2.4(2.2, 2.7) | 2.5(2.2, 2.7) | 22.2(21.4, 23.0) | 72.9(72.0, 73.8) |  | 2.2(2.0, 2.5) | 1.9(1.7, 2.1) | 23.0(22.1, 23.9) | 72.9(71.8, 74.0) |
| 9th | 2.2(1.9, 2.4) | 2.1(1.9, 2.3) | 17.9(17.3, 18.6) | 77.8(77.1, 78.6) |  | 1.9(1.6, 2.2) | 1.5(1.3, 1.7) | 19.8(18.8, 20.8) | 76.8(75.6, 78.0) |
| **Sex** |  |  |  |  |  |  |  |  |  |
| Boys | 3.1(2.8, 3.3) | 2.6(2.4, 2.9) | 19.6(19.0, 20.1) | 74.7(74.0, 75.4) |  | 2.7(2.5, 2.9) | 1.7(1.5, 1.9) | 19.2(18.5, 20.0) | 76.3(75.4, 77.3) |
| Girls | 2.1(1.9, 2.3) | 2.1(1.9, 2.2) | 22.8(22.1, 23.6) | 73.0(72.2, 73.8) |  | 1.6(1.5, 1.7) | 1.8(1.6, 2.0) | 25.9(24.9, 26.9) | 70.7(69.5, 71.9) |
| **Residence** |  |  |  |  |  |  |  |  |  |
| Urban | 2.4(2.2, 2.6) | 2.1(1.9, 2.3) | 19.3(18.5, 20.1) | 76.2(75.4, 77.1) |  | 1.9(1.7, 2.0) | 1.4(1.3, 1.5) | 20.4(19.5, 21.3) | 76.4(75.4, 77.3) |
| Rural | 2.7(2.4, 3.0) | 2.5(2.3, 2.7) | 21.8(21.1, 22.5) | 73.0(72.2, 73.8) |  | 2.4(2.1, 2.6) | 2.0(1.7, 2.3) | 23.4(22.3, 24.6) | 72.2(70.7, 73.8) |
| **Region** |  |  |  |  |  |  |  |  |  |
| North | 1.9(1.7, 2.1) | 2.0(1.6, 2.3) | 22.0(20.3, 23.7) | 74.2(72.2, 76.1) |  | 1.7(1.5, 1.9) | 1.5(1.2, 1.9) | 23.4(21.8, 25.0) | 73.4(71.4, 75.3) |
| East | 2.3(2.0, 2.5) | 1.8(1.5, 2.1) | 18.9(17.9, 19.8) | 77.1(76.1, 78.0) |  | 1.9(1.7, 2.2) | 1.3(1.1, 1.5) | 19.7(18.4, 21.0) | 77.0(75.5, 78.6) |
| Central | 2.0(1.7, 2.4) | 2.0(1.7, 2.2) | 21.3(19.9, 22.7) | 74.7(73.1, 76.3) |  | 1.8(1.5, 2.0) | 1.5(1.2, 1.8) | 21.0(19.8, 22.3) | 75.7(74.3, 77.1) |
| South | 3.5(2.8, 4.1) | 3.7(3.3, 4.1) | 25.2(23.3, 27.0) | 67.7(66.0, 69.4) |  | 3.0(2.6, 3.4) | 2.3(2.0, 2.7) | 26.0(23.8, 28.2) | 68.7(66.2, 71.2) |
| Southwest | 3.1(2.6, 3.6) | 2.8(2.3, 3.4) | 20.8(19.4, 22.3) | 73.3(71.4, 75.1) |  | 2.8(2.2, 3.4) | 2.4(1.5, 3.4) | 25.2(21.7, 28.6) | 69.6(64.9, 74.3) |
| Northwest | 2.9(1.9, 3.9) | 2.3(1.9, 2.8) | 20.7(19.3, 22.2) | 74.0(72.3, 75.7) |  | 2.5(1.5, 3.5) | 1.8(1.3, 2.2) | 20.0(18.9, 21.2) | 75.7(73.5, 77.9) |
| Northeast | 2.4(2.0, 2.8) | 2.2(1.9, 2.5) | 20.8(19.2, 22.3) | 74.7(72.9, 76.5) |  | 2.0(1.7, 2.3) | 2.2(1.8, 2.5) | 23.8(22.2, 25.5) | 72.0(70.1, 73.9) |
| **GDP per capita category** |  |  |  |  |  |  |  |  |  |
| Low | 2.5(2.2, 2.8) | 2.7(2.3, 3.0) | 24.1(22.9, 25.2) | 70.7(69.4, 72.1) |  | 2.4(2.1, 2.7) | 2.1(1.8, 2.3) | 25.3(24.2, 26.4) | 70.2(68.9, 71.5) |
| Middle | 2.5(2.2, 2.8) | 2.1(1.9, 2.3) | 19.5(18.7, 20.4) | 75.9(74.9, 76.8) |  | 2.1(1.8, 2.4) | 1.9(1.4, 2.3) | 22.0(20.3, 23.6) | 74.1(71.8, 76.3) |
| High | 2.7(2.3, 3.0) | 2.4(2.1, 2.7) | 20.6(19.6, 21.5) | 74.3(73.3, 75.4) |  | 2.1(1.9, 2.3) | 1.4(1.2, 1.6) | 20.4(19.3, 21.5) | 76.1(74.8, 77.3) |
| **Current cigarette smoking** |  |  |  |  |  |  |  |  |  |
| No | 2.4(2.2, 2.6) | 2.1(2.0, 2.3) | 20.8(20.2, 21.3) | 74.7(74.1, 75.3) |  | 2.0(1.9, 2.2) | 1.5(1.4, 1.7) | 22.0(21.2, 22.8) | 74.4(73.5, 75.4) |
| Yes | 4.2(3.6, 4.7) | 5.3(4.7, 5.9) | 26.4(24.8, 28.1) | 64.1(62.5, 65.8) |  | 5.3(4.2, 6.4) | 7.2(6.2, 8.1) | 30.4(28.6, 32.2) | 57.1(55.4, 58.8) |

Data are presented as %(95%CI).
